# Genomic characterization and gene bank curation of *Aegilops*: the wild relatives of wheat

**DOI:** 10.3389/fpls.2023.1268370

**Published:** 2023-10-17

**Authors:** Laxman Adhikari, John Raupp, Shuangye Wu, Dal-Hoe Koo, Bernd Friebe, Jesse Poland

**Affiliations:** ^1^ Plant Breeding and Genetics Lab, Center for Desert Agriculture, Biological and Environmental Science and Engineering Division, King Abdullah University of Science and Technology (KAUST), Thuwal, Saudi Arabia; ^2^ Wheat Genetics Resource Center, Department of Plant Pathology, Kansas State University, Manhattan, KS, United States; ^3^ Plant Science Program, Biological and Environmental Science and Engineering Division, King Abdullah University of Science and Technology (KAUST), Thuwal, Saudi Arabia

**Keywords:** *Aegilops*, genotyping-by-sequencing (GBS), gene bank curation, genetic diversity, phylogenetic analysis, population structure, wheat wild relatives

## Abstract

Genetic diversity found in crop wild relatives is critical to preserve and utilize for crop improvement to achieve sustainable food production amid climate change and increased demand. We genetically characterized a large collection of 1,041 *Aegilops* accessions distributed among 23 different species using more than 45K single nucleotide polymorphisms identified by genotyping-by-sequencing. The Wheat Genetics Resource Center (WGRC) *Aegilops* germplasm collection was curated through the identification of misclassified and redundant accessions. There were 49 misclassified and 28 sets of redundant accessions within the four diploid species. The curated germplasm sets now have improved utility for genetic studies and wheat improvement. We constructed a phylogenetic tree and principal component analysis cluster for all *Aegilops* species together, giving one of the most comprehensive views of *Aegilops*. The *Sitopsis* section and the U genome *Aegilops* clade were further scrutinized with in-depth population analysis. The genetic relatedness among the pair of *Aegilops* species provided strong evidence for the species evolution, speciation, and diversification. We inferred genome symbols for two species *Ae*. *neglecta* and *Ae*. *columnaris* based on the sequence read mapping and the presence of segregating loci on the pertinent genomes as well as genetic clustering. The high genetic diversity observed among *Aegilops* species indicated that the genus could play an even greater role in providing the critical need for untapped genetic diversity for future wheat breeding and improvement. To fully characterize these *Aegilops* species, there is an urgent need to generate reference assemblies for these wild wheats, especially for the polyploid *Aegilops*.

## 1 Introduction

Global climate change with increasingly variable weather, declining soil quality, and increased biotic and abiotic stresses impede crop production. For instance from crop modeling, an increase in a global mean temperature of a degree Celsius reduces the global wheat yield by 6% (Asseng et al., 2015; Zhao et al., 2017). In this context, the continual genetic improvement of commercial cultivars is needed, including incorporating novel alleles for improved stress tolerance and disease resistance. However, the domestication bottleneck and variety selection practices are major drivers that limit the genetic diversity currently available in the primary gene pool for wheat (*Triticum aestivum* L.) improvement (Haudry et al., 2007). Several studies have indicated that wild wheat relatives are reliable sources for increasing the genetic diversity in wheat breeding (Lopes et al., 2015; Leigh et al., 2022; Ahmed et al., 2023).

The genus *Aegilops* encompasses the secondary and tertiary gene pool of bread wheat with a central role in wheat evolution and domestication being the donors of B and D subgenomes. The *Aegilops* species are critically important in providing biotic resistance and abiotic tolerance as well as yield-related genetic loci to wheat (Kishii, 2019; Rakszegi et al., 2020). For instance, *Ae*. *speltoides* harbors agronomically important genes, such as *Sr32* which is effective against the devastating wheat stem rust pathogen Ug99 (Friebe et al., 1996). Similarly, *Ae*. *kotschyi* has been shown to confer leaf and stripe rust resistance with genes *Lr54* and *Yr37* (Marais et al., 2005), and *Ae*. *biuncialis* possesses a wheat powdery mildew resistance gene (Li et al., 2019). Likewise, the 2NS translocation from *Ae*. *ventricosa* provided multiple disease resistance including root-knot nematode, stripe rust, stem rust, leaf rust, and the wheat blast caused by *Magnaporthe oryzae* (Cruz et al., 2016; Gao et al., 2021). Finally, *Ae*.*tauschii* has been frequently used in wheat breeding as the genetic resource for various wheat disease resistance and abiotic-stress tolerance (Suneja et al., 2019).

Although *Aegilops* species hold great potential as genetic resources, limited information is available on the genomic characterization of the genus as a whole. Most of the work to date has focused on a limited number of *Aegilops* species and has been based on cytology, traditional molecular markers, and a limited number of loci. Genomic characterization is complex, because *Aegilops* species have various ploidy levels and unique genomic compositions and some polyploids have multiple copies of the same sub-genome [e.g., DDM, 6X *Ae*. *crassa*]. Also, reference genomes for only a few *Aegilops* species have been released to date. Therefore, the complicated genomic features and inadequate resources are major challenges for *Aegilops* population studies and more focused, targeted mining of the genetic resources.

These limitations are quickly changing with the recently available genome assemblies of some diploid *Aegilops* such as *Ae*. *tauschii* (Luo et al., 2017), *Ae*. *speltoides* and *Ae*. *longissima* (Avni et al., 2022), *Ae*. *sharonensis* (Yu et al., 2022), *Ae*. *bicornis*, and *Ae*. *searsii* (Li et al., 2022). These genome assemblies are shedding light on *Aegilops’* evolutionary and population genetic analysis. Additionally, the high-throughput sequencing method such as genotyping-by-sequencing (GBS), which can generate *de*-*novo* genomics variants for complex genome species (Poland et al., 2012), has also been proven as an efficient genotyping tool for gene bank collections (Adhikari et al., 2022a).

The Wheat Genetics Resource Center (WGRC) gene bank at Kansas State University has been maintaining myriads of wild wheat accessions under the *Triticum* and *Aegilops* genera. We previously curated the collections of A-genome diploid wheat (Adhikari et al., 2022a) and *Ae*. *tauschii* (Singh et al., 2019a). Thus, the focus of this current study was to characterize the genetic diversity, population structure, and genomic composition of the *Aegilops* collection in the WGRC with the curation of the germplasm. Throughout this study, we followed the *Aegilops* species nomenclature by Van Slageren (1994) except for *Ae*. *mutica*, and genome symbols were followed as described by Waines and Barnhart (1992). Utilizing variants from GBS, we dissected the genetic and genomic relationships among the 23 *Aegilops* species through phylogenetic clustering, principal component analysis (PCA), population structure analysis, and diversity analysis. We also examined *Aegilops* and wheat genomes relationships through *Aegilops* sequence mapping to the wheat genome and genetic clustering.

## 2 Materials and methods

### 2.1 Plant resources

**Figure 1:**
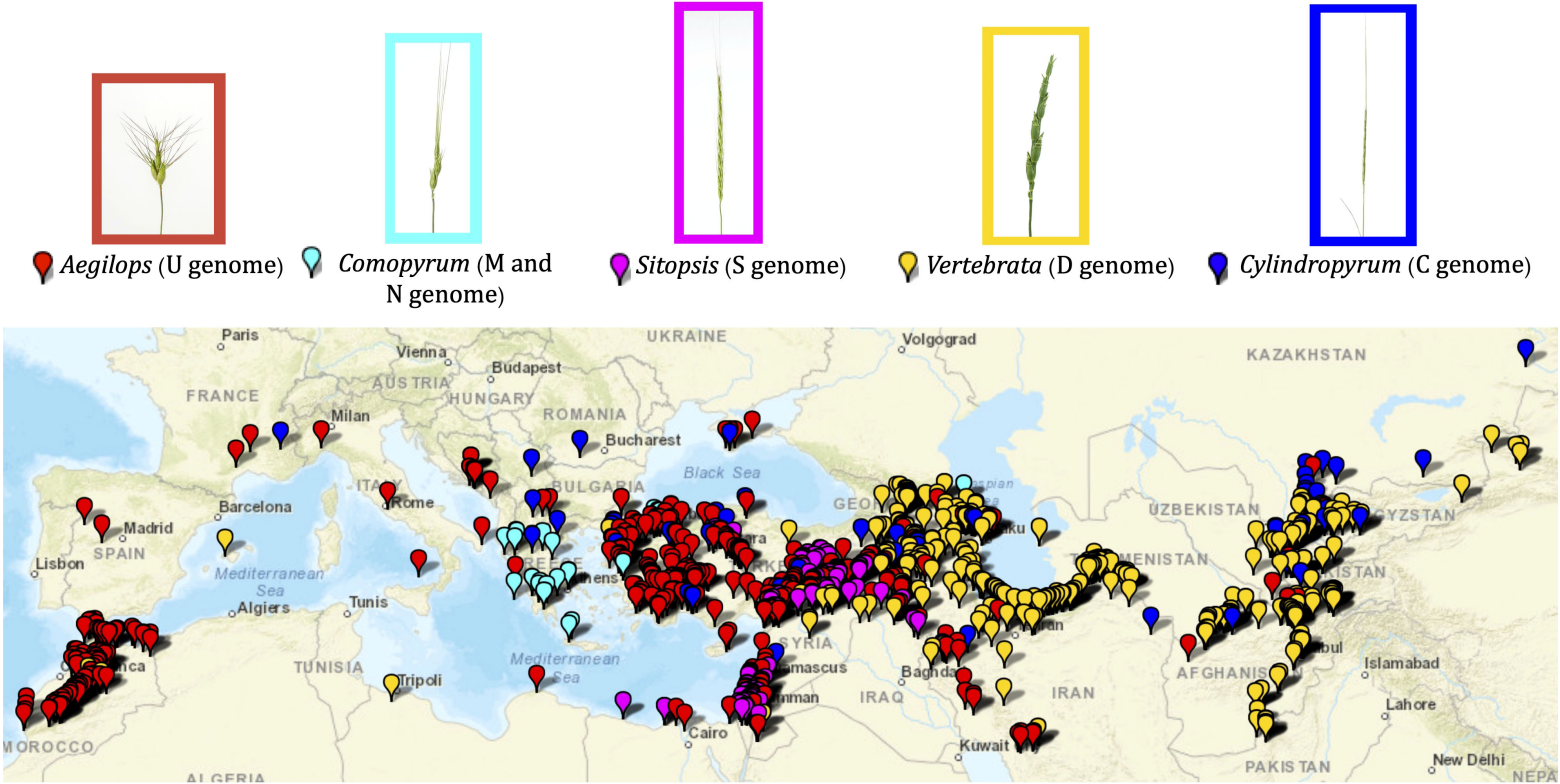
Geographic distribution of the *Aegilops* accessions maintained in the WGRC gene bank. Spike morphologies of representative accessions for the five *Aegilops* sections are shown with the enclosed rectangles. Each section is designated by corresponding color.

This study primarily included 1,041 accessions of the *Aegilops* species preserved and maintained in the WGRC gene bank (**Supplementary Material Table S1**; **Figure 1**). The accessions were originally collected from various sources and sites including the Middle East, Anatolia, East Asia, and northern Africa (**Figure 1**; **Supplementary Material Table S1**). Accessions comprise 22 different *Aegilops* species under five sections (*Aegilops*, *Comopyrum*, *Cylindricum*, *Sitopsis*, and *Vertebrata*) (Van Slageren, 1994) and *Ae*. *mutica*, which is synonymously known as *Amblopyrum muticum*. For gene bank curation and most part of the population analysis, only those *Ae*. *tauschii* accessions that were not in the previous gene bank curation experiment (Singh et al., 2019a) were used. We also used CIMMYT wheat lines and already curated *Ae*. *tauschii* lines (**Supplementary Material Table S1**) for genotyping together with the diploid *Aegilops* to dissect the genetic relationships among wheat and *Aegilops* genomes.

Most of these species are self-pollinated and were primarily maintained by single seed descent, with exceptions described below. *Ae*. *speltoides* and *Ae*. *mutica* are partially out-crossing and were maintained through sib-mating multiple plants. Specifically, *Ae*. *mutica* accessions consisted of 54 samples from five out-crossing plants bulked together.

### 2.2 Genotyping and marker identification

The DNA extraction, GBS library preparation, and sequencing were performed as we described in our earlier studies (Adhikari et al., 2022a) using two enzyme-based GBS (Poland et al., 2012). Only a single plant per accession was sequenced for all species except *Ae*. *mutica*, where we sequenced 54 individuals obtained from randomly crossing five plants, because the species is cross-pollinating and it has a low germination rate.

For the *de*-*novo* single nucleotide polymorphism (SNP) calling, reads were demultiplexed using sabre (https://github.com/najoshi/sabre) and adapters were trimmed using fastp (Chen et al., 2018). The variants were called using the available reference assemblies of diploid *Aegilops* and wheat and using mock references generated as described (Melo et al., 2016; Adhikari et al., 2018). For mock references, the raw GBS reads of selected accessions with higher sequence data were used as the reference source. We also ensured that the mock reference represents the sequences of relevant *Aegilops* species or the genomes [C, D, M, N, S, U, T] for the population to be genotyped. The *de*-*novo* variants were called using BCFtools (Li, 2011) and used for initial gene bank curation and population clustering of the whole collection. Then the *de*-*novo* variants were also called for some species independently depending on the objectives of the specific analysis (**Supplementary Material Table S2**). For some species in polyploid lineages, we called variants on a diploid ancestor and, later, the same variants were called in the polyploids using BCFtools (Li, 2011). After calling variants, unless otherwise stated, we filtered loci to keep any variants passing these conditions: minor allele frequency (MAF) >0.01, missing <30%, and heterozygous <10%.

The TASSEL5 GBSv2 pipeline was used for reference-based SNP calling (Glaubitz et al., 2014). For this method, *Ae*. *tauschii* reference genome Aet v5.0 (Wang et al., 2021) or *Ae*. *sharonensis* (Yu et al., 2022), *Ae*. *speltoides* (Avni et al., 2022), *Ae*. *searsii*, and *Ae*. *bicornis* (Li et al., 2022) genomes were used. We also called variants in all these diploids species to the wheat reference using the “Chinese Spring” wheat reference (IWGSC CS RefSeq v2.1) (Zhu et al., 2021) to observe the relationship between *Aegilops* and wheat.

### 2.3 Gene bank curation

In the first step, the germplasm curation identified misclassified accessions and corrected the taxonomy of these accessions in the database (Singh et al., 2019a). We identified misclassified accessions by constructing a phylogenetic cluster colored with the recorded species. These were further verified using PCA clustering followed by a visual assessment of seeds and spikes. The misclassified accessions were identified and confirmed with multiple genotyping sets *viz.* entire collection, species alone, and same genome accessions together.

In the second step, the genetically identical accessions were determined using allele matching (Singh et al., 2019a; Adhikari et al., 2022a). However, this assessment was done only for the accessions of the species whose reference genome is available, for example, *Ae*. *tauschii* and the *Sitopsis* section *Aegilops*. The allele matching (>99% identity by state) was used as a threshold to confirm genetically identical accessions. Allele matching used homozygous and non-missing sites between two given accessions, and the raw markers were filtered using MAF >0.01, missing <50%, and heterozygous <20% parameters before allele matching. We conducted further examinations of the sets of genetic duplicates to assess their phenotypic similarities, collection sites, and sources of collection.

### 2.4 Genetic clustering, populationanalysis, and diversity

The genotyping matrices were analyzed for the genetic distances among the *Aegilops* populations, which were then used for exploring the population structure and ancestry. For phylogenetic clustering, the genetic distance was computed using the “dist” function in R (R Core Team, 2020), and the R packages *ape* (Paradis and Schliep, 2019) and *phyclust* (Chen, 2011) were then used to generate unrooted neighbor-joining (NJ) tree with the default parameters (Singh et al., 2019b; Adhikari et al., 2022a).

The genetic relationships among the *Aegilops* accessions were further examined via PCA, which was performed in two steps. The A matrix was derived from A.mat() function within the R package rrBLUP (Endelman, 2011), and the eigenvalues and eigenvectors were derived using the “e” function (Adhikari et al., 2022a). Furthermore, the population structure of the *Sitopsis* group of *Aegilops* was also performed with the reference-based genotyping profile using fastStructure software (Raj et al., 2014) as explained (Adhikari et al., 2022a). We computed Nei’s diversity index (Nei, 1987) and total segregating loci for each of the *Aegilops* species to assess the relative diversity of the species.

### 2.5 *Ae*. *columnaris* and *Ae*. *neglecta* genome symbols

We investigated the traditional genome symbols of *Ae*. *columnaris* (UM) and *Ae*. *neglecta* (UM, UMN) for the presence/absence of the M genome. There are recent cytology-based findings that have questioned the traditional genome symbols of these species (Badaeva et al., 2018). To test this, we computed the sequence read mapping and segregating loci on the M and U mock reference genomes for the *Ae*. *columnaris* and *Ae*. *neglecta* accessions as well as two other tetraploids (*Ae*. *nelglecta* and *biuncialis*) whose genomic compositions are unequivocally recognized as MU or UM. The *de*-*novo* variants were first identified for the diploid M genome (*Ae*. *comosa*) and U genome (*Ae*. *umbellulata*) populations separately, and then the same variants were called on these four tetraploid species. We also constructed the phylogenetic clustering among *Ae*. *columnaris*, *Ae*. *neglecta*, *Ae*. *geniculata*, *Ae*. *biuncialis*, and a tetraploid that shares only the U genome (*Ae*. *triuncialis*) to see their relative positions in the tree.

### 2.6 The *Aegilops* genome relation to the wheat genome

We mapped diploid *Aegilops* GBS reads to the wheat genome (CS.Ref.v1) (Appels et al., 2018) and computed sequence read mapping coverage. The reads mapped per Mb wheat subgenome and the total variants mapped for each wheat subgenome (A, B, D) were recorded. We did not further evaluate *Ae*. *tauschii* whose close genetic relationship as the wheat D subgenome donor has been clearly established. We also generated an unrooted NJ phylogenetic tree among diploid *Aegilops* and wheat using the variants called on wheat B and D reference subgenomes independently.

## 3 Results

### 3.1 *Aegilops* distributions

*Aegilops* species characterized in this study were primarily collected around the Fertile Crescent, Anatolia, central Asia, northern Africa, and southern Europe (**Figure 1**; **Supplementary Material Table S1**). Of the five sections, the *Aegilops* section [*Ae*. *umbellulata* (U), *Ae*. *kotschyi* (US), *Ae*. *peregrina* (US), *Ae*. *triuncialis* (CU), *Ae*. *columnaris* (UM), *Ae*. *biuncialis* (UM), *Ae*. *neglecta* (UM, UMN), *Ae*. *geniculata* (MU)] exhibited a much wider distribution from central Asia to northern Africa (**Figure 1**). The species of *Cylindropyrum* [*Ae*. *markgraffii* (C), *Ae*. *caudata* (C), and *Ae*. *cylindrica* (CD)] were primarily collected from Uzbekistan, Tajikistan, Kazakhstan, Azerbaijan, and Turkey. The species of the *Comopyrum* [*Ae*. *comosa* (M), *Ae*. *uniaristata* (N)] mainly come from Greece, Turkey, and Russia. The *Sitopsis* (S genome) species [*Ae*. *bicornis*, *Ae*. *searsii*, *Ae*. *sharonesis*, *Ae*. *longissima*, and *Ae*. *speltoides*] were predominantly collected in Turkey, Israel, Syria, Iraq, and Jordan. The *Vertebrata* section species [*Ae*. *tauschii* (D), *Ae*. *crassa* (DM, DDM), *Ae*. *ventricosa* (DN), *Ae*. *juvenalis* (DMU), and *Ae*. *vavilovii* (DMS)] were obtained from central Asia to southern Europe (**Figure 1**; **Supplementary Material Table S1**). The *Ae*. *mutica* tested here originated from Turkey and Armenia (**Supplementary Material Table S1**).

### 3.2 Marker discovery

We identified 54,667 *de novo* called SNPs for the entire *Aegilops* collections genotyped together. After filtering (MAF >0.01, missing <30%, and heterozygosity <10%), we retained 46,879 SNPs (**Table 1**). We removed 10 accessions (TA2674, TA2633, TA1733, TA11097, TA1740, TA2178, TA2042, TA1739, TA2316, and TA2296) with high rate of missing call (>80%). When we separated the genotyping information per species, we identified filtered segregating SNPs in the range of 1,483 for *Ae*. *searsii* to 14,322 for *Ae*. *speltoides* (**Table 1**). We also generated other SNP-genotyping matrices for analysis-specific purposes, such as for particular species’ genetic relations and for genetically identical accession determination (**Supplementary Material Table S2**).

**Table 1:** *Aegilops* species with number of accessions, number of segregating loci, and the Nei’s diversity indices.

Species	# Accessions	Segregating loci	Nei’s index
All collection	1041	54667	0.104
*Ae*. *tauschii*	47	3369	0.024
*Ae*. *vavilovii*	6	9955	0.093
*Ae*. *mutica*	54*	8094	0.053
*Ae*. *ventricosa*	17	5828	0.05
*Ae*. *uniaristata*	24	5416	0.019
*Ae*. *umbellulata*	58	3391	0.015
*Ae*. *triuncialis*	199	8601	0.032
*Ae*. *speltoides*	97	14322	0.072
*Ae*. *sharonensis*	9	2224	0.019
*Ae*. *searsii*	18	1483	0.013
*Ae*. *peregrina*	33	7981	0.053
*Ae*. *neglecta*	71	11931	0.062
*Ae*. *markgrafii*	16	3474	0.022
*Ae*. *longissima*	14	3043	0.023
*Ae*. *kotschyi*	24	6876	0.053
*Ae*. *juvenalis*	9	8796	0.081
*Ae*. *geniculata*	143	8248	0.038
*Ae*. *cylindrica*	79	6173	0.046
*Ae*. *crassa*	32	8999	0.074
*Ae*. *comosa*	17	3388	0.025
*Ae*. *columnaris*	12	5382	0.041
*Ae*. *biuncialis*	52	7819	0.042
*Ae*. *bicornis*	13	1493	0.012

(*) The *Ae. mutica* being cross-pollinated we used many different samples from a single accession (s), so total of 54 plants rather than accessions.

### 3.3 Gene bank curation

#### 3.3.1 Misclassified accessions

**Figure 2:**
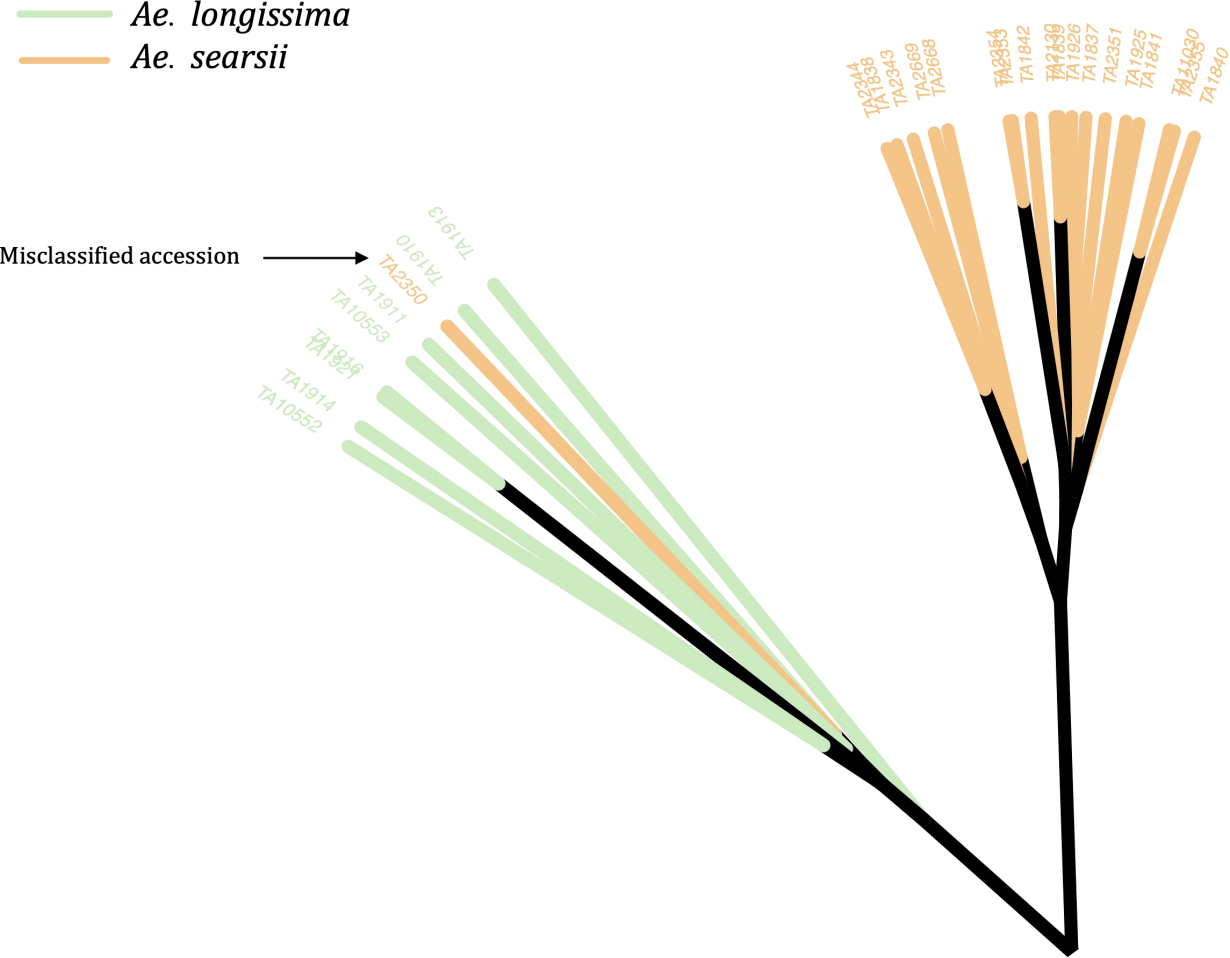
An unrooted neighbor-joining (NJ) tree with an example of a misclassified accession (TA2350) in the WGRC gene bank. The genetically clustered clades were colored based on the morphological classes of the accessions and visually accessed. The misclassified accession TA2350, which was previously grouped under *Ae*. *searsii* (orange clade) was re-classified as *Ae*. *longissima* (green).

The phylogenetic clustering and PCA enabled us to identify and correct the classification of 49 accessions (**Figure 2**; **Supplementary Material Table S3**). Most of the misclassified accessions were observed within tetraploid *Aegilops*. Twelve accessions that were previously considered as *Ae*. *triuncialis* were now identified as different *Aegilops*, whereas nine accessions that were classified as different *Aegilops* species are now re-identified as *Ae*. *triuncialis* (**Supplementary Material Table S3**). Similarly, 11 accessions identified as *Ae*. *neglecta* were now genetically identified as different *Aegilops*. The other misclassified example includes four accessions of each of *Ae*. *geniculata* and *Ae*. *vavilovii* (**Supplementary Material Table S3**). A few misclassified accessions of diploid *Aegilops* included *Ae*. *umbellulata* (2), *Ae*. *markgrafii* (2), and *Ae*. *searsii* (1) (**Figure 2**). The classes of all misclassified accessions were updated prior to the downstream population genomic analysis.

#### 3.3.2 Genetically identical accessions

The gene bank curation discovered total 28 genetically identical accessions in *Ae*. *tauschii* and four members of the *Sitopsis* section (**Supplementary Material Table S3**). There were no pairs of *Ae*. *speltoides* accessions that have allele matching above 95%. Of 28 duplicated accessions, 17 were from *Ae*. *tauschii*, even though we only had a total of 47 *Ae*. *tauschii* accession for this experiment (**Supplementary Material Table S3**). In our previous study, we also reported many genetically identical accessions in *Ae*. *tauschii* collection (Singh et al., 2019a). The gene bank curator’s observations also confirmed the phenotypic similarities among these genetically proven duplicate *Aegilops* accessions. As we examined the sources of these duplicate accessions, we found that most of them come from various institutes rather than from direct collectors. For instance, the *Ae*. *bicornis* genetically identical accessions TA1952, TA1956, and TA11023 were obtained from Kyoto University, the University of Manitoba, and the University of Missouri, respectively (**Supplementary Material Table S1**).

### 3.4 Phylogenetic clustering, PCA, and population structure

**Figure 3:**
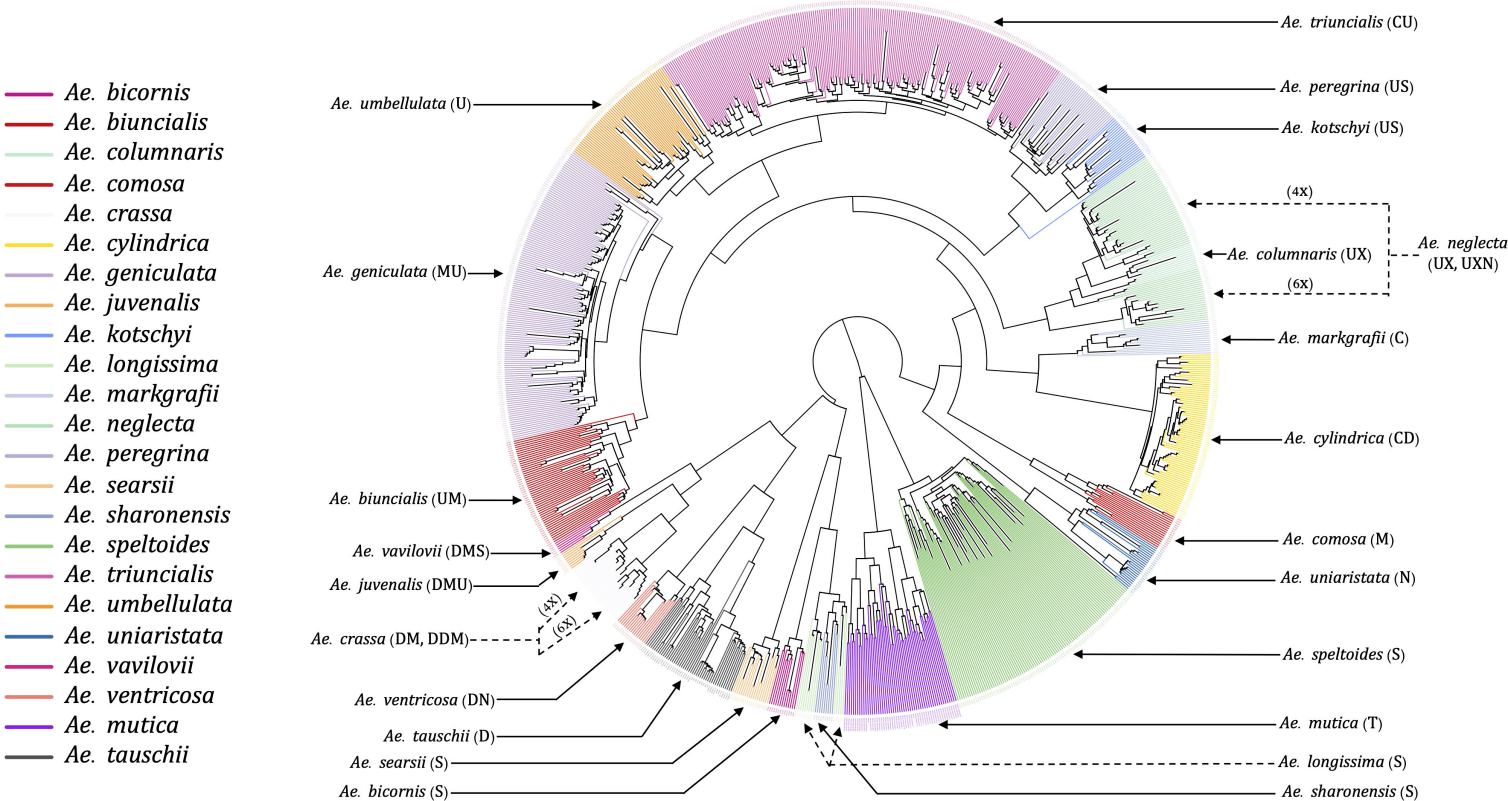
An unrooted neighbor-joining (NJ) tree of 23 different *Aegilops* species. The tree branches were colored based on the accessions genetic grouping after adjusting the misclassified accessions classes. The genome signs of each of the species were annotated along with their names as indicated by solid and dotted arrowheads.

The unrooted NJ phylogenetic tree with all tested *Aegilops* accessions gave clear separation of species as the branches of clades and sub-clades differentiated all 23 species and the relevant groups (**Figure 3**). We observed the species sharing genomes as closely related clades, such as *Ae*. *kotschyi* and *Ae*. *peregrina* (SU) and *Ae*. *geniculata* and *Ae*. *biuncialis* (UM), clustered into respective primary clades. Overall, there were three primary clades: (i) the first clade consisted of *Ae*. *speltoides* and *Ae*. *mutica*; (ii) the second clade has four diploids of *Sitopsis* (except *Ae*. *speltoides*), *Ae*. *tauschii*, and D genome polyploids (except *Ae*. *cylindrica*); (iii) the third primary clade has all other species, including M, N, C, and U genome diploids and polyploids.

The hexaploid (6X) and tetraploid (4X) species within a clade, such as *Ae*. *neglecta* and *Ae*. *crassa*, were grouped separately by ploidy. The ploidy levels of these genetically clustered sub-groups (6X and 4X) were also verified using chromosome counting (**Supplementary Material Figure S1**) following Koo et al. (2017). The chromosome numbers of some accessions of *Ae*. *crassa* (**Supplementary Material Figure S2**) were also confirmed with the published data (Badaeva et al., 1998).

**Figure 4:**
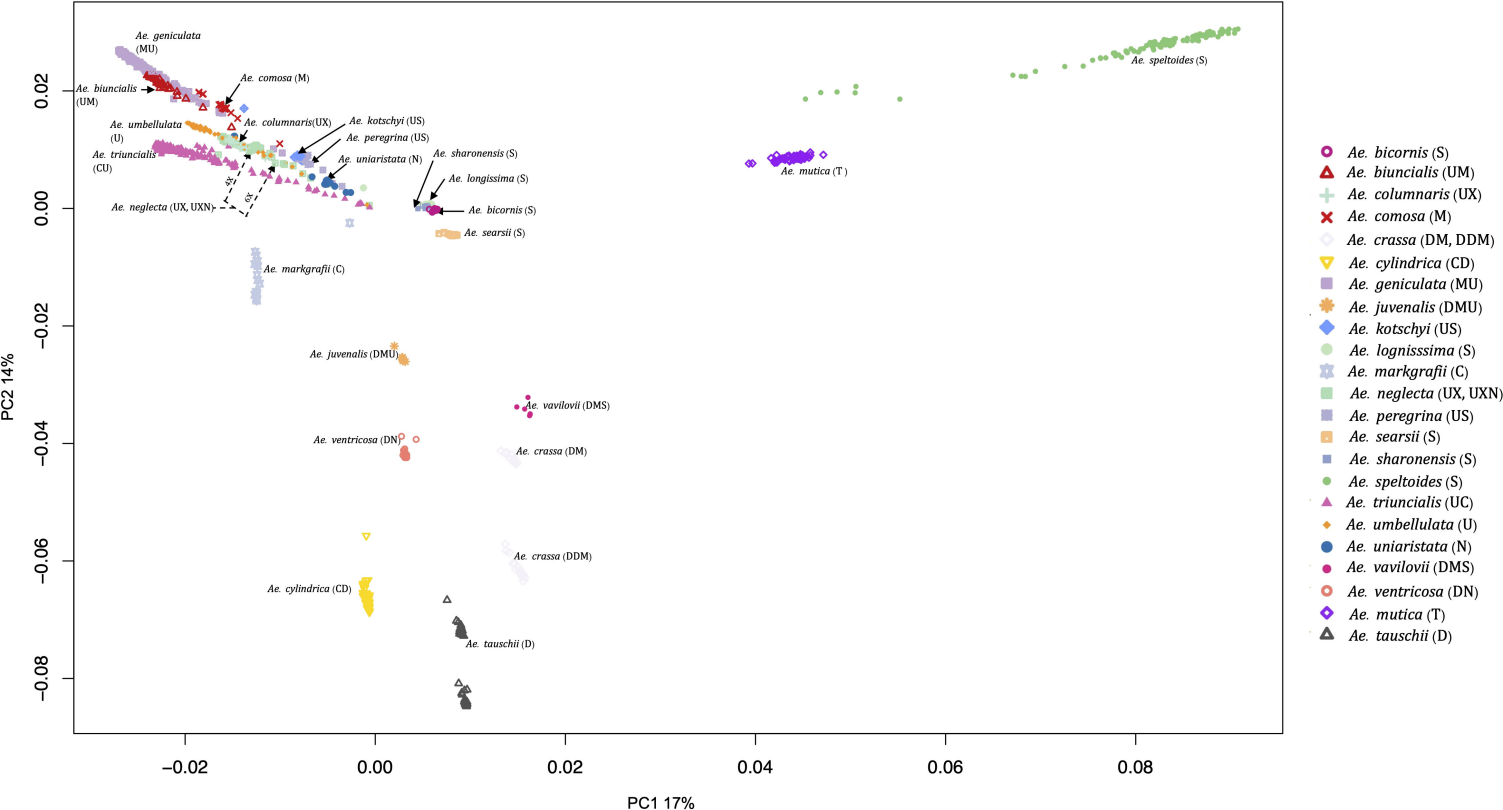
Principal component analysis (PCA) plot for all 23 *Aegilops* species with the first PCs. The 23 *Aegilops* species were grouped and colored based on their species and genome compositions.

PCA also grouped the *Aegilops* species commensurate with the phylogenetic analysis. The first and second principal components (PC1 and PC2) explained about 17% and 14% of the variations among the *Aegilops*, respectively. PC1 separated *Ae*. *speltoides* from other polyploids and diploids (**Figure 4**), while the PC2 primarily differentiated *Ae*. *tauschii* and *Ae*. *speltoides*, the D genome donor to wheat and the potential sister group of the wheat B genome donor, respectively. As in phylogenetic analysis, PCA grouping also divided the 4X and 6X accessions of the *Ae*. *neglecta* and *Ae*. *crassa* (**Figure 4**).

### 3.5 Population genomics of *Sitopsis* and *Ae*. *mutica*

**Figure 5:**
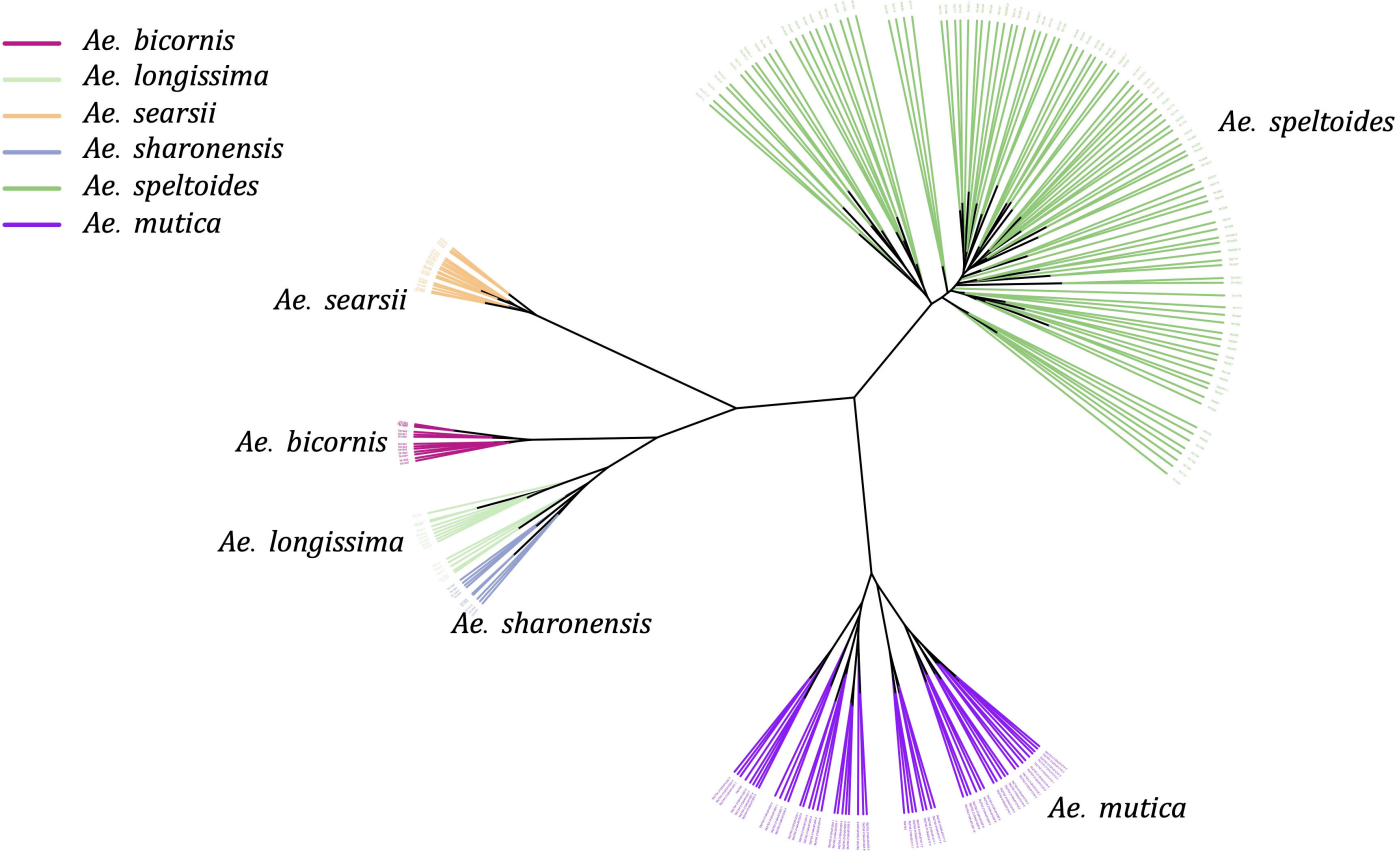
An unrooted Neighbor-Joining tree of five *Aegilops* species including *Sitopsis* section members (S genome) and *Ae*. *mutica* (T genome).

As we observed the separation of four *Sitopsis* members with *Ae*. *speltoides* and *Ae*. *mutica*, we separately examined the population of these species using reference-based variants from the *Ae*. *speltoides* genome assembly. The constructed phylogenetic tree distinctly divided the S-genome diploids into two large clades, one representing *Ae*. *speltoides* and the other with the remaining four *Sitopsis* (**Figure 5**). The genetic clustering corresponded to the historical sub-section division of the section is *Truncata* (*Ae*. *speltoides*) and the *Emarginata*. We also observed that the *Ae*. *mutica* (T genome) clustered closer to *Ae. speltoides* both in PCA and phylogenetic analysis (**Figure 5**). The relationships among *Sitopsis* group and *Ae*. *mutica* were further verified by computing pairwise Nei’s F_ST_ (Nei, 1987), where we observed *Ae*. *mutica* has the closest genetic relationship [lowest F_ST_ (0.65)] with *Ae*. *speltoides*, closer than any other members of the *Sitopsis* (**Supplementary Material Table S4**). Hence, all these analyses support that *Ae*. *mutica* as the sister taxon to *Ae*. *speltoides* and it is an *Aegilops* species.

Furthermore, within the S-genome diploids, the *Ae*. *speltoides* and *Ae*. *searsii* had the most genetic differentiation with the highest F_ST_ value 0.88 (**Supplementary Material Table S4**). However, the pairwise F_ST_ indicated that *speltoides* is genetically almost equally and highly differentiated from all other S-genome diploids (*Emarginata*) (**Supplementary Material Table S4**).

**Figure 6:**
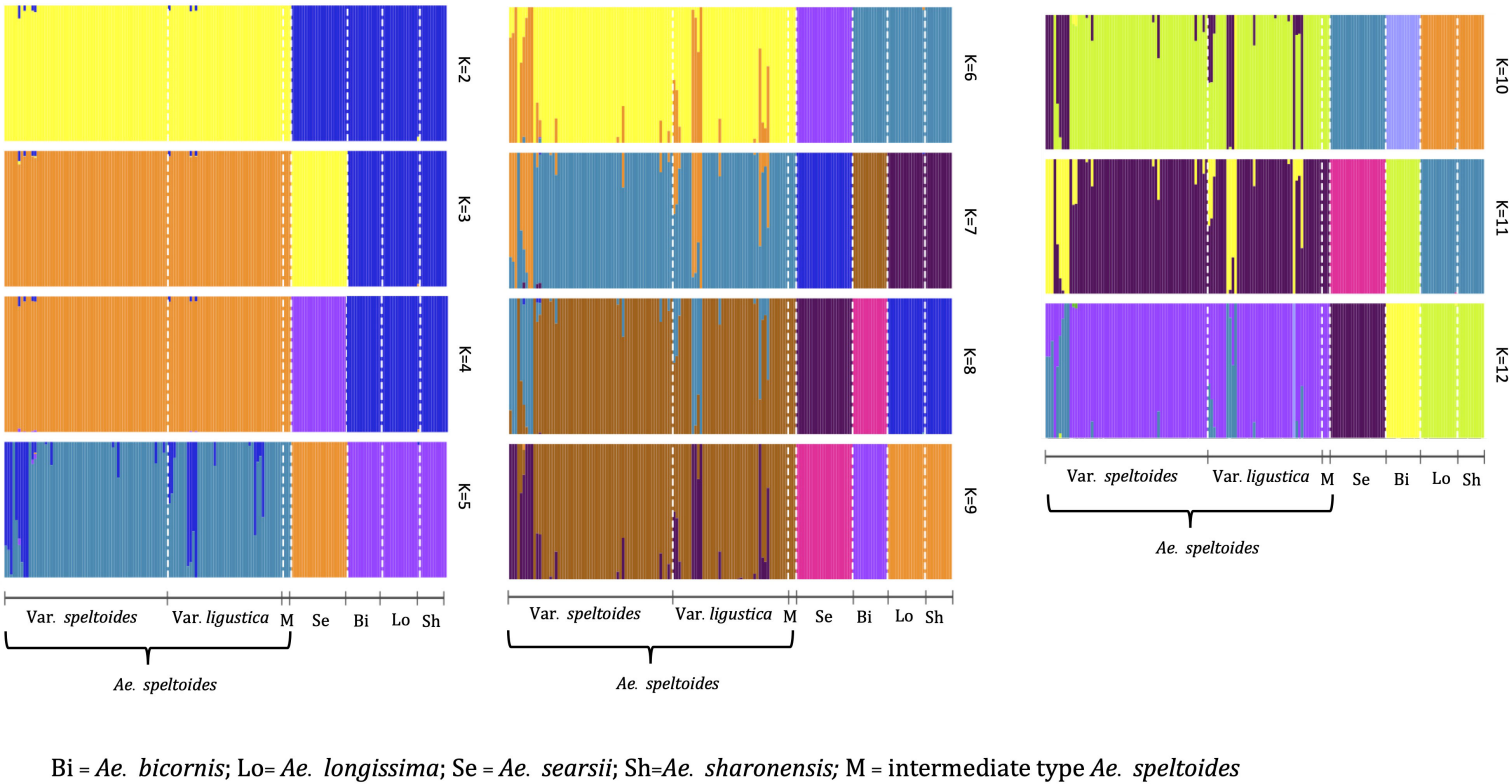
The population structure of S-genome diploids *Aegilops*, where the value of K and colors of the bars indicate the description of the groups. Each color represents a population and each bar with more than one color indicates the admixtures with the admixture proportions as represented by the proportion of each color.

Population structure analysis of S-genome diploids matched with the phylogenetic tree and pairwise F_ST_ analysis. At *K* = 2, there was a differentiation between *Ae*. *speltoides* and the rest of the *Sitopsis*, while at *K* = 3, *Ae*. *searsii* also differentiated from the rest of the *Sitopsis* (**Figure 6**). At *K* = 7, *Ae*. *bicornis* accessions separated from others and then no new differentiation was observed until *K* = 12. Both in the phylogenetic tree and in population structure analysis, the *Ae*. *longissima* and *Ae*. *sharonensis* appeared as highly genetically similar groups (**Figures 5**, **6**). In fact, there was no population differentiation between these two species at any level of K. The pairwise F_ST_ values also confirmed that these two species have the lowest pairwise F_ST_ = 0.006 (**Supplementary Material Table S4**), and the population differentiation is very low. Furthermore, two sub-groups within *Ae*. *speltoides*, var. *speltoides*, and var. *ligustica* also did not differentiate at any levels of K in the population structure analysis (**Figure 6**) and the PCA (**Supplementary Material Figure S3**). However, within *Ae*. *speltoides*, a few admixtures were observed and were differentiated for their geographical origins (**Figure 6**).

### 3.6 *Ae*. *umbellulata* and U-genome tetraploids

**Figure 7:**
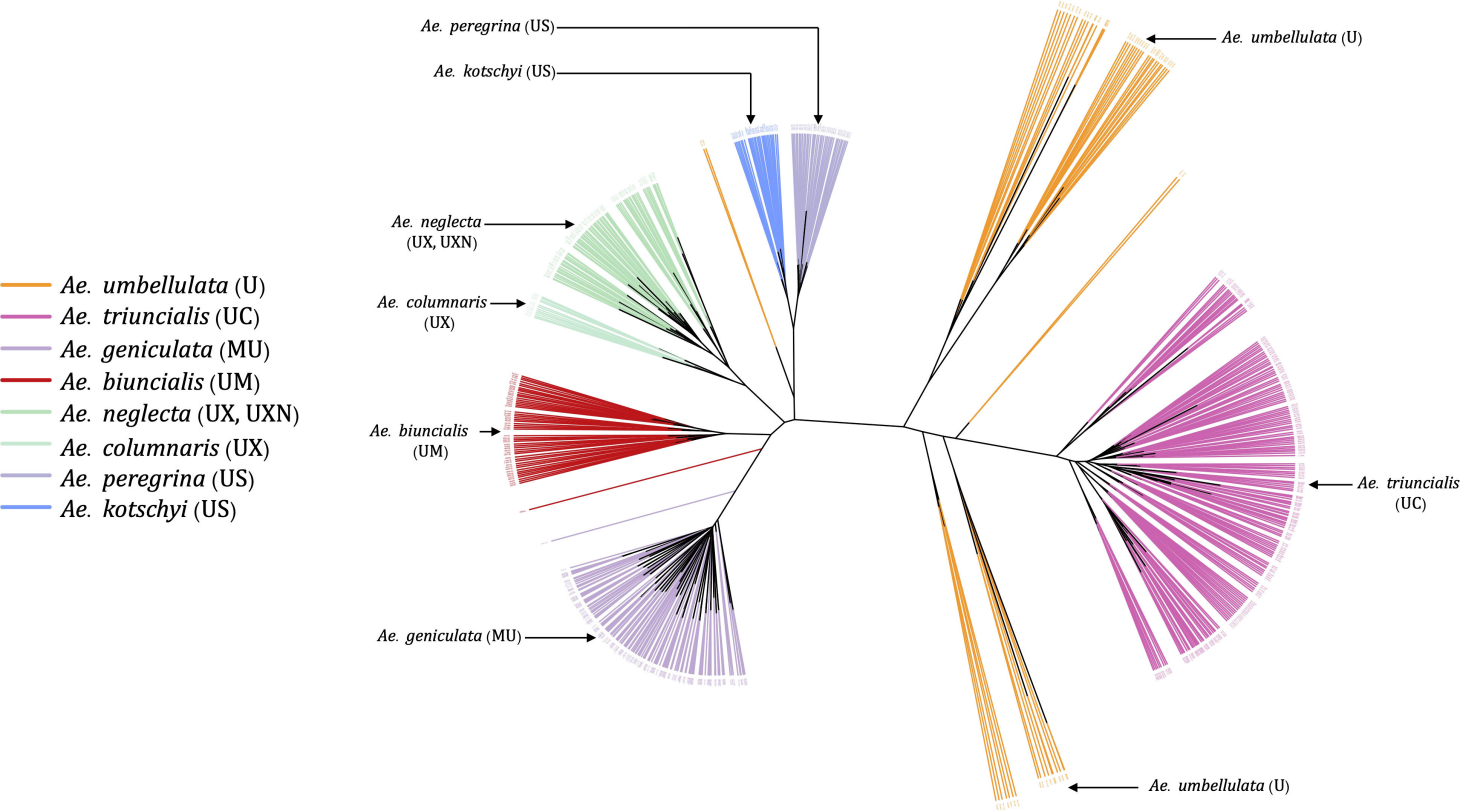
An unrooted neighbor-joining (NJ) tree for *Ae*. *umbellulata* and U genome containing tetraploids within the genus *Aegilops*.

Most of the tetraploid *Aegilops* have the U genome; therefore, understanding the genetic relationship among members of the U-genome clade gives insight into a large set of taxa in the genus. Phylogenetic clustering of these species only showed two larger clades, where one was represented by *Ae*. *triuncialis* (UC) and the other had all remaining tetraploids (**Figure 7**). The diploid *Ae*. *umbellulata* sits on the intermediate position between the larger clades. Although the variants were only called on U-genome (*Ae*. *umbellulata*) *de-novo* reference, the tetraploids distinctly grouped for their genomic compositions. The tetraploid species *Ae*. *pregerina* and *Ae*. *kotschyi* (US genome), *Ae*. *neglecta* and *Ae*. *columnaris* (traditionally assigned as UM), and the UM genome tetraploids *Ae*. *biuncialis* and *Ae*. *geniculata* formed a separate clade and sub-clades (**Figure 7**). Also, we observed the splitting of *Ae*. *umbellulata* accessions into smaller clades. With a few exceptions as noted below, these phylogenies largely agree with previous genome designations.

### 3.7 Genome symbols of *Ae*. *columnaris* and *Ae*. *neglecta*

*Ae*. *columnaris* and *Ae*. *neglecta* formed a different clade than the other tetraploids with U and M genomes such as *Ae*. *geniculata* (UM) and *Ae*. *biuncialis* (MU) in both phylogenetic clustering and PCA (**Figures 3**, **4**, **7**; **Supplementary Material Figure S4**). The comparative positions of these tetraploids with other tetraploids in the genetic cluster indicated that these two tetraploids must be given unique genome symbols than the *Ae*. *geniculata* and *Ae*. *biuncialis* (**Supplementary Material Figure S4**). Thus, we hypothesized that *Ae*. *columnaris* and *Ae*. *neglecta* do not carry the M genome. The absence of M genome in *Ae. columnaris* and *Ae. neglecta* accessions was further confirmed by computing total reads mapped and total variants called on M-genome (*Ae*. *comosa* mock reference) and U genome (*Ae*. *umbellulata* mock reference) (**Supplementary Material Figure S5**, **Supplementary Material Table S5**). All four tetraploid species, namely, *Ae. columnaris* and *Ae. neglecta* along with *Ae. geniculata* and *Ae. biuncialis* exhibited an equal percentage of overall reads alignment (~38%) on the U genome, whereas the percentage read alignment of *Ae. columnaris* and *Ae. neglecta* on M genome was low (~21%) as compared to the alignment of *Ae. geniculata* and *Ae. biuncialis* reads (~38%). We also noticed that a few *Ae*. *comosa* segregating loci were mapped for *Ae*. *columnaris* (10%) and *Ae*. *neglecta* (24%) on the M genome. In contrast, *Ae*. *biuncialis* had 50% and *Ae*. *geniculata* had 46% M-genome loci. Hence, the proportion of mapped reads and loci also suggested that the *Ae. neglecta* and *Ae*. *columnaris* must have the U genome, but a different second sub-genome than M. Thus, we proposed that *Ae. columnaris* and *Ae. neglecta* genome formulas are most likely UX (X, the unknown genome) or UXN in hexaploid form as proposed based on the cytology (Dvorak, 1998; Badaeva et al., 2018).

### 3.8 *Aegilops* species diversity

For the entire collection, we obtained 54,667 SNPs, which were skewed to low MAF as expected for a diverse population like this (**Supplementary Material Figure S6**). Despite the differences in population size, the total segregating loci for the species or groups were mostly dependent on the ploidy levels and the reproductive biology (inbreed vs. outcrossing) (**Table 1**). The polyploids and out-crossing species had a higher number of segregating loci compared to other diploids (**Table 1**). Notably, the MAF of the loci in partially cross-pollinated species, such as *Ae*. *speltoides*, had a higher frequency (**Supplementary Material Figure S7**) than that of the MAF of the loci for the entire *Aegilops* collection (**Supplementary Material Figure S6**).

The Nei’s diversity indices also followed the pattern of segregating loci which were greater in polyploid and cross-pollinated species. We computed Nei’s diversity index for the entire collection as 0.10 (**Table 1**). Of all 23 species, *Ae*. *bicornis* had the lowest Nei’s diversity index (0.012) followed by *Ae*. *searsii* (0.013) and *Ae*. *umbellulata* (0.015). Among the diploids, the *Ae*. *speltoides* had the highest Nei’s diversity (0.072), which was followed by *Ae*. *mutica* (0.053). Among the tetraploids, the *Ae*. *triuncialis* had the lowest diversity index (0.032) while the *Ae*. *neglecta* had the highest diversity index (0.062). The hexaploid species *Ae*. *vavilovii* has the highest Nei’s diversity index value among all 23 species analyzed in the experiment (**Table 1**). This increased diversity can be attributed to various factors such as multiple gene copies, hybridization during speciation, increased mutation rates, and more opportunities for recombination due to the presence of multiple genomes.

### 3.9 Wheat and *Aegilops* genomes

**Figure 8:**
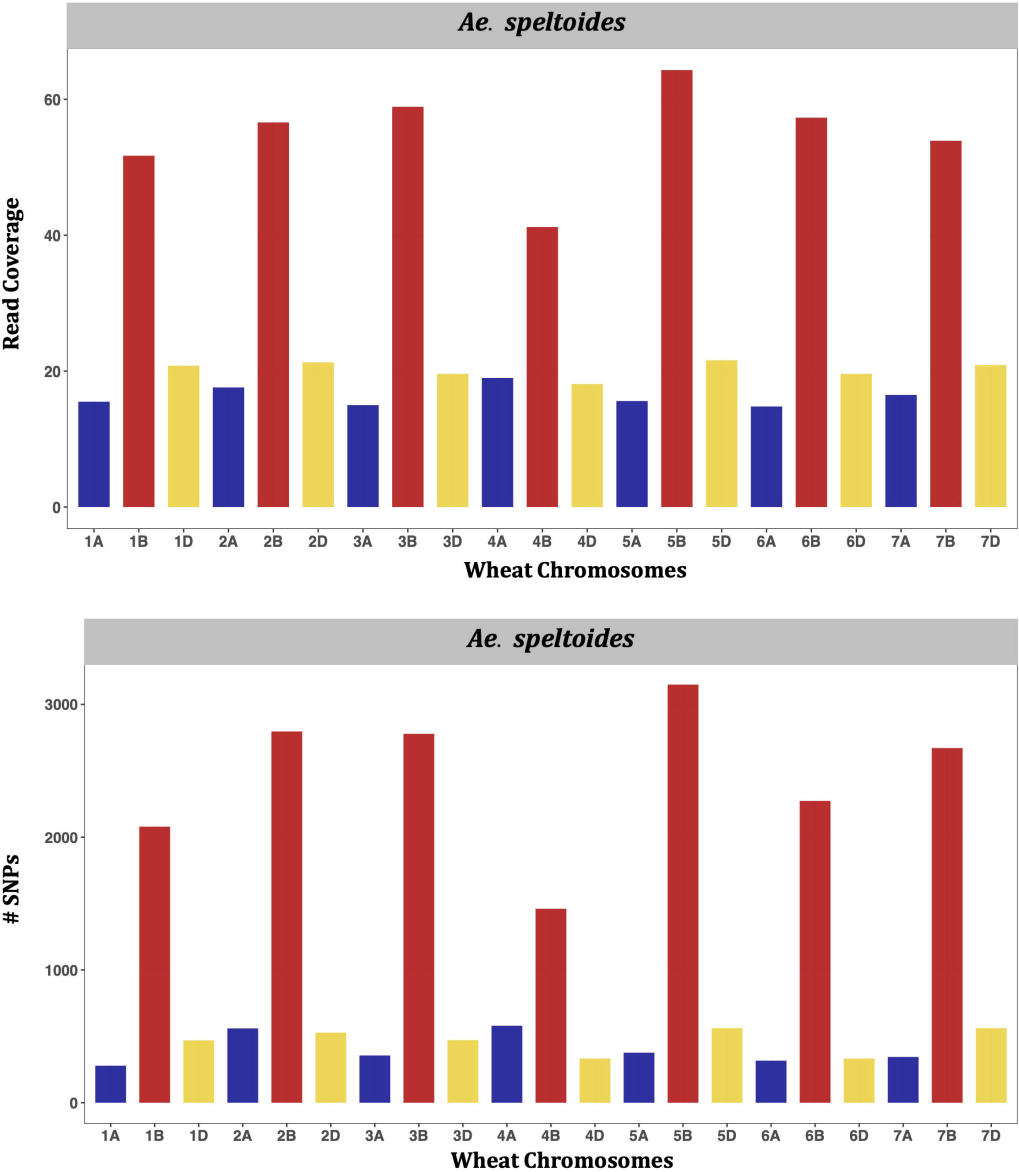
Bar charts showing genomic relations between *Ae*. *speltoides* and wheat. The average number of *Ae*. *speltoides* sequence reads mapped per Mb of the wheat genome (upper panel), and numbers of *Ae*. *speltoides* variants mapped on the respective wheat chromosomes (lower panel).

The genetic clustering between wheat and all diploid *Aegilops* showed that *Ae*. *tauschii* is the nearest extant *Aegilops* to the bread wheat (**Supplementary Material Figure S8**). The genetic cluster clearly showed that *Ae*. *speltoides* is not closer to wheat as *Ae*. *tauschii* and even other diploids, and supporting that, *Ae*. *speltoides* is likely not the direct progenitor of the wheat subgenome B (**Supplementary Figure S8**). However, the *Ae*. *speltoides* read depth mapping and SNP detection occurred at its maximal on the wheat subgenome B (**Figure 8**), indicating the species as the sister group of wheat B genome progenitor. Furthermore, the other members of the *Sitopsis* group clustered between *Ae*. *speltoides* clade and the clade with *Ae*. *tauschii* and the wheat subclades in the phylogenetic tree (**Supplementary Material Figure S8**). Consistent with the genetic clustering, their maximum read mapping and SNP detection also occurred at subgenome D and B chromosomes (**Supplementary Material Figures S8**–**S10**), suggesting that the four members of *Sitopsis*, except *Ae*. *speltoides*, have very strong genomic relationships with both D and B subgenomes.

Similarly, in the U genome diploid (*Ae*. *umbellulata*), the highest proportion of sequence reads was mapped onto wheat chromosomes of the D subgenome, followed by those of the A and B subgenomes (**Supplementary Material Figure S11**). Exceptionally, a slightly higher proportion of reads were mapped on 2A than the 2D. The pattern of SNP detection was exactly the same as read mapping, indicating that wheat subgenome D is the closest to the U genome of the *Aegilops*. However, relations between the wheat A genome and the *Aegilops* U genome cannot be overlooked, as reasonably higher reads and loci were mapped on the A genome as compared to the wheat B genome (**Supplementary Material Figure S11**). Likewise, the highest number of reads and SNPs were mapped onto wheat subgenome D for the N genome diploid (*Ae*. *uniaristata*) (**Supplementary Material Figure S12**), for the M genome diploid (*Ae*. *comosa*) (**Supplementary Material Figure S13**), and C genome diploid (*Ae*. *markgraffii*) (**Supplementary Material Figure S14**). These observations suggest that the N, M, and C genomes of *Aegilops* are also genetically closer to the D subgenome than A and B.

Interestingly, the *Ae*. *mutica* accessions when mapped onto the wheat subgenomes showed higher sequence read and loci mapped on the wheat D subgenome (**Supplementary Material Figure S15**). The read and loci mapping pattern was unchanged even when we replaced wheat D subgenome chromosomes with *Ae*. *tauschii* chromosomes. Nevertheless, all types of population grouping within *Aegilops* (**Figures 3** **–** **5**; **Supplementary Material Figure S8**) evidently showed that *Ae*. *mutica* is a sister group of *Ae*. *speltoides* and still a member of B lineage. Some recent studies based on whole genome sequencing data have also reported a higher sequence read and loci mapping of *Ae*. *mutica* on the wheat D subgenome compared to others (Grewal et al., 2022; Li et al., 2022).

## 4 Discussions

### 4.1 Multi-species diverse *Aegilops* collection and gene bank curation

In this study, we genotyped over a thousand accessions representing almost all species of the *Aegilops* genus, covering the full range of their natural distributions under the Van Slageren (1994) nomenclature, with missing only *Ae*. *caudata*. We curated the WGRC gene bank *Aegilops* collection, giving curated germplasm sets that are ready to screen for the novel alleles and utilize in the breeding program. The misclassified accession were confirmed with multiple analyses including phylogenetic clustering of the whole population, species or genome-specific populations and PCA, therefore there is strong support for the genotype-based identification of these misclassified accessions (**Supplementary Material Table S3**). Since the genotype-based clustering evidently differentiated the hexaploid and tetraploid accessions within the species such as *Ae*. *crassa* and *Ae*. *neglecta*, we can also provide the ploidy levels information as a means of within-species classification and update the gene bank database.

Here, we identified the redundant accessions in the species with variants called directly on reference genome assemblies. This gives increased power and accuracy in variant calling. Therefore, we suggest the re-assessment of genetically redundant accessions for other *Aegilops* species in the future when reference assemblies are available. For the polyploid *Aegilops*, reference variant calling can be done whenever the component species reference genomes are available using a combined reference genome or independent variant calling to each genome. As we examined the origins of these genetically verified and visually confirmed duplicates, we discovered that many of them originated from various research institutes rather than directly from collectors. Therefore, we here recommend the need for curating the global collection of these naturally collected germplasms, as the same genetic materials can be preserved under different plant IDs or accession numbers. In our previous studies, we also observed several duplicates originating from the exact same collection sites (Singh et al., 2019a; Adhikari et al., 2022a). This is because these self-pollinated species have already reached genomic saturation, and the progeny of the same mother parents are genetically identical inbred. Although we do not suggest discarding the duplicated accessions identified here, we strongly suggest for considering these results when utilizing the collection, such as screening the accessions for disease resistance or developing introgression populations. Overall, gene bank curation helps in the management, preservation, and utilization of the germplasms (Singh et al., 2019a; Volk et al., 2021).

### 4.2 *Aegilops* population analysis

This is the most comprehensive *Aegilops* population genetic study reported so far with over 45 thousand *de*-*novo* filtered SNPs and reference-based variants. In the study, we took advantage of recently completed chromosome-scale genome assemblies of diploid *Aegilops* (Wang et al., 2021; Avni et al., 2022; Li et al., 2022; Yu et al., 2022). Until now, the lack of genomic resources including reference assemblies has been a major issue hindering the species population genomic analysis. Therefore, future genomic studies on *Aegilops* must focus on generating more genomic resources for other diploids and polyploids. With a larger population and thousands of genomic variants, the population grouping that we observed here was at the finest level, enabling us to differentiate the 4X and 6X accessions within a species (**Supplementary Material Figure S1**).

### 4.3 *Ae. speltoides*, other *Sitopsis* and *Ae. mutica*

Our genetic analysis supports that the *Ae*. *mutica* requires no genus-level separation from other *Aegilops* as Van Slageren (1994) suggested. It is genetically an *Aegilops* taxon closer to *Ae*. *speltoides* (**Figures 4**, **5**). This is in agreement with recent reports (Bernhardt et al., 2020; Li et al., 2022). Further genomic analysis may require high coverage genomic data and a greater number of samples to better understand the relationship among *Ae*. *mutica* and other diploid *Aegilops*. Additionally, the genetic differences that we observed here between the *Truncata* (*Ae*. *speltoides*) and *Emarginata* (four other) *Sitopsis* were greater; therefore, the redefinition of the section *Sitopsis* could be desirable. One of the ideas could be the separation of *Ae*. *speltoides* from the rest of the four *Sitopsis* members and regrouping the *Ae*. *speltoides* with *Ae*. *mutica* (**Figures 3** **–** **5**; **Supplementary Material Figure S8**).

We also showed that the *Ae*. *sharonensis* and *Ae*. *longissima* have very high genetic similarities or a low genetic differentiation (F_ST_ = 0.006) and are most likely the sub-species of the same species. Also, both of these species are equally distant from *Ae*. *speltoides*. The finding is also supported by the latest study, where Avni et al. (2022) reported that the genomes of these two species are highly similar with identical genome sizes and also share 292 orthogroups.

In this study, we observed a little genetic difference between the two sub-taxa of *Ae*. *speltodies*; var. *speltoides* and *ligustica* with no population differentiation (**Figure 6**; **Supplementary Material Figure S3**), in accordance with several past studies. These two sub-groups of *speltoides* not only have distinct spike morphology and mode of seed dispersal but also exhibit similar karyotype structure, producing fully fertile hybrid and mixed stands of two types naturally exhibits (Zohary and Imber, 1963). A single locus *Lig* on chromosome 3S governs the spike morphology of these two sub-groups (Luo et al., 2005); otherwise, they are highly genetically similar.

### 4.4 U-genome species, some tetraploid genome symbols and polyploid *Aegilops*

The U genome tetraploids and its progenitor *Ae*. *umbellulata* genetic clustering revealed the unique relationships among the species. We observed the *Ae*. *umbellulata* accessions split into sub-groups in such a way that some accessions were clustered closer to *Ae*. *triuncialis* clade whereas some other accessions reposed near the other tetraploid clades (**Figure 7**), suggesting the potential unique *Ae*. *umbellulata* ancestries for the two groups.

In this study, we found further evidence that the *Ae*. *columnaris* and *Ae*. *neglecta* genome symbols should not include the M genome designation (**Supplementary Material Figures S4**, **S5** and **Supplementary Table S5**), based on sequence read and loci mapping data, and phylogenetic clustering (**Supplementary Material Figure S4**). Cytology-based approaches (Resta et al., 1996; Dvorak, 1998; Badaeva et al., 2004; Badaeva et al., 2018) have previously discussed this issue and suggested the symbol “X” (Resta et al., 1996). Several lines of evidence, including low chromosome pairing in hybrids of *Ae*. *columnaris* x *Ae*. *comosa* (the M genome progenitor), variation in repetitive nucleotide sequences, and differences in the karyotype structure C-banding pattern, have been used to confirm the absence of the M genome in *Ae*. *neglecta* and *Ae*. *columnaris* (Badaeva et al., 2018). This study has provided further verification with thousands of loci. Therefore, we suggest research communities for the consistent use of genome symbols for *Ae. columnaris* (UX) and *Ae*. *neglecta* (UX or UXN). Furthermore, cytological and genomic evaluation of the X genome is certainly warranted.

### 4.5 *Aegilops* genetic diversity

Ploidy level and the mode of fertilization appeared as major determinants of *Aegilops* accessions diversity (**Table 1**). Interestingly, we did not observe the direct impact of population size on Nei’s diversity index (Nei, 1987) at any ploidy levels (**Table 1**). For example, the diploid *Ae*. *sharonensis* (nine accessions) exhibited a higher diversity index (0.019) compared to *Ae*. *umbellulata* (58 accessions), and the tetraploid *Ae*. *ventricosa* (17 accessions) had a higher diversity index than another tetraploid, *Ae*. *triuncialis* (199 accessions) (**Table 1**). Additionally, we noted that *Ae*. *speltoides*, as the diploid species, displayed the greatest diversity, and relatively higher diversity indices were observed in the S genome polyploids such as *Ae*. *kotschyi*, *Ae*. *peregrina*, and *Ae*. *vavilovii* (**Table 1**). In summary, most of the *Aegilops* species exhibited a wider and more variable diversity and had greater potential to be utilized in wheat breeding. Therefore, it is crucial to make serious efforts toward the *in-situ* conservation of these germplasms and enhance *ex-situ Aegilops* germplasm collections. Kilian et al. (2011) also emphasized the urgency of protecting these *Aegilops* germplasms, highlighting the importance of understanding *Aegilops* genetic diversity, *Aegilops*-*Triticum* molecular biological relationships, and identifying and preserving suitable *Aegilops* alleles for wheat breeding.

### 4.6 *Aegilops* and wheat genomes

This study represents, perhaps, the first comprehensive report on genomic relationships between all *Aegilops* genomes and wheat sub-genomes, based on high-throughput sequence-based markers and robust phylogeny of these wild wheat species. Consistent with some earlier reports, our findings indicate that most of the *Aegilops* genomes (U, M, N, C) are genetically closer to the wheat D subgenome (**Supplementary Material Figures S9**-**S15**), with the exception of *Ae*. *speltoides* (**Figure 8**). Several studies have reported that the speciation event of the B genome donor occurred earlier than the speciation of *Ae*. *tauschii* (the D-genome lineage), resulting in stronger evolutionary relationships of the U, M, N, and C diploid *Aegilops* within the D-genome lineage (Glémin et al., 2019; Tanaka et al., 2020; Said et al., 2021).

In our study, we observed unique relationships between certain genomes within the *Aegilops*-*Triticum* complex that had not been clearly described in earlier studies. One of the most important observations is that four *Sitopsis* species exhibit relationships with both the B and D subgenomes of wheat. These relationships were evident in the phylogenetic tree and supported by statistic on sequence read and mapped loci (**Supplementary Material Figures S8**-**S10**). Interestingly, recent reports have also considered these four *Sitopsis* members as part of the D lineage, and are closer to the wheat D subgenome (Li, 2011; Avni et al., 2022; Li et al., 2022).

### 4.7 *Ae*. *mutica*, wheat genomes, and homoploid hybridization

In this study, we observed unique genetic characteristics of *Ae*. *mutica* as it was phylogenetically closer to the *Ae*. *speltoides* (**Figures 3** **–** **5** and **Supplementary Material Figure S8**); however, it showed genetic similarities with the wheat D subgenome (**Supplementary Material Figure S15**). Interestingly, similar observations have been reported in recent studies. Li et al. (2022) reported lower genetic similarities between *Ae*. *mutica* and wheat B subgenome computed as genetic relatedness. Likewise, Grewal et al. (2022) reported a similar relationship between *Ae*. *mutica* and wheat subgenomes, with the highest number of *Ae*. *mutica* loci mapped on the D subgenome, rather than the A and B subgenomes (**Supplementary Material Figure S15**). Therefore, the genetic similarities and phylogenetic relationship between the *Ae*. *mutica* and the *Aegilops*-*Triticum* complex are exclusive and warrant further investigation in a larger population with high-depth sequencing. Furthermore, these analyses indicate that *Ae*. *mutica* genome may have undergone independent evolution or played a role in the evolution of polyploid genomes following its divergence from *Ae*. *speltoides*. Some recent studies also argued that *Ae*. *mutica* and the D lineage underwent homoploid hybridization followed by introgression (Bernhardt et al., 2020; Li et al., 2022). Bernhardt et al. (2020) reported that most of the members of the *Aegilops* genus, except *Ae*. *speltoides*, likely evolved through ancient primordial hybrid speciation events involving the ancestral *Triticum* and *Ae*. *mutica*. Earlier studies also indicated a higher degree of homology between *Ae*. *mutica* and the wheat D subgenome (Jones and Majisu, 1968).

### 4.8 Utilizing *Aegilops* novel alleles in high-throughput genotyping era

This study establishes a solid foundation for the future utilization of *Aegilops* germplasm within the WGRC gene bank. The development of introgression populations, combined with new genomic tools, has the potential to accelerate the selection and advancement of novel alleles in wheat breeding. In an ongoing investigation, we have successfully created wheat—*Ae*. *speltoides* introgression lines and have achieved the mapping of introgression segments using a skim-sequencing approach (Adhikari et al., 2022b). Likewise, association genomics approaches can be leveraged to identify novel *Aegilops* alleles directly within the wild germplasm collections (Gaurav et al., 2022). As an example, candidate genes associated with various agronomic traits in another wild wheat relative, einkorn, were identified using the cost-effective skim-sequencing technique (Saripalli et al., 2023). Within this context, the importance of these highly diverse *Aegilops* accessions is further enhanced. Finding trait-related alleles through genome-wide association studies, generating reference assemblies, and resequencing diverse panels represent some of the future steps in harnessing the potential of these valuable *Aegilops* genetic resources for enhancing wheat.

In conclusion, this study has unveiled the genomic and genetic relationships among all *Aegilops* species and demonstrated the efficient use of the GBS approach for curating gene bank accessions and investigating the genetic diversity and population structure of the entire *Aegilops* collection. Most likely this is the first genomic analysis of a nearly complete set of the genus *Aegilops* encompassing 23 species. We dissected a larger population (1,041) using over 45K SNPs and constructed a robust phylogenetic tree and the PCA clusters. The population grouping and structuring of this valuable wild wheat species largely align with the traditional nomenclatures at the species level. Moreover, using these high-throughput genome-wide markers, we have confirmed the genome symbols of two tetraploid species that were previously under debate in the literature.

Our findings also reveal that each *Aegilops* subgenome and wheat subgenomes exhibit unique relationships at the genomic level, warranting further investigation. Notably, *Ae*. *mutica* showed unique characteristics, appearing as a sister group of *Ae*. *speltoides*, yet displaying a higher number of sequences and variants mapped onto the wheat subgenome D. The genetic and evolutionary relationships among *Aegilops* and with wheat will become clearer when we have more genomic resources, such as genome assemblies and resequencing data for each *Aegilops* species. This study offers a comprehensive view of the relative genetic diversities of all 23 species together for the first time. The substantial genetic diversity observed, along with its relative extent in each *Aegilops* species, presents an opportunity to select species and germplasms as sources of novel alleles for wheat breeding and improvement.

## Data availability statement

The Raw GBS data, the fastq files, are available in the Sequence Read Archive (SRA) of the National Center for Biotechnology Information (NCBI) under the BioProject accession PRJNA985892. The key file and necessary SNP matrices and the R script files (.rmd) are provided in the dryad public repository which are available with the unique DOI: 10.5061/dryad.mgqnk994n. All data are available in the article or the supplementary files and at the Dryad digital repositories https://datadryad.org/stash/dataset/doi:10.5061/dryad.mgqnk994n.

## Author contributions

LA: Data curation, Formal Analysis, Investigation, Methodology, Validation, Visualization, Writing – original draft, Writing – review & editing. JR: Data curation, Formal Analysis, Methodology, Resources, Validation, Writing – review & editing. SW: Investigation, Methodology, Writing – review & editing. D-HK: Conceptualization, Formal Analysis, Investigation, Methodology, Resources, Validation, Visualization, Writing – review & editing. BF: Conceptualization, Methodology, Resources, Supervision, Validation, Writing – review & editing. JP: Conceptualization, Funding acquisition, Investigation, Methodology, Project administration, Resources, Supervision, Validation, Writing – original draft, Writing – review & editing.

## Supplementary material

Supplementary Figure 1 The GBS SNP–based unrooted neighbor-joining (NJ) tree separating tetraploid and hexaploid accessions of *Ae*. *neglecta* (blue clade) and the chromosome counts of two representative individuals from each 4X and 6X sub-clade of the *Ae*. *neglecta*.

Supplementary Figure 2 An unrooted neighbor-joining (NJ) tree of *Ae*. *juvenalis*, *Ae*. *crassa*, *and Ae*. *vavilovii.* The tree branches were colored based on the accession’s taxon.

Supplementary Figure 3 Principal component analysis (PCA) plot showing two forms of *Ae. speltoides*: var. *speltoides* and *ligustica*.

Supplementary Figure 4 An unrooted neighbor-joining (NJ) tree separating some tetraploid *Aegilops* accessions containing two species whose genome formula is controversial, the *Ae*. *neglecta* and *Ae*. *columnaris*.

Supplementary Figure 5 The bar chart showing the overall sequence read alignment of four tetraploid *Aegilops* species: *Ae. biuncialis*, *Ae*. *geniculata*, *Ae*. *columnaris*, and *Ae*. *neglecta* when aligned on M and U genome *de*-*novo* mock reference.

Supplementary Figure 6 Minor allele frequency (MAF) distribution within the loci for the entire *Aegilops* collection.

Supplementary Figure 7 Distribution of minor alleles frequency (MAF) for segregating variants in *Ae*. *speltoides*.

Supplementary Figure 8 An unrooted neighbor-joining (NJ) tree constructed using the genotyping information generated by using wheat B genome as a reference (left); and the unrooted NJ tree constructed using genotyping profile generated using the wheat D genome as a reference (right).

Supplementary Figure 9 Bar charts showing genomic relations between the *Sitopsis* section *Aegilops* (except *Ae*. *speltoides*) and the wheat.

Supplementary Figure 10 Bar charts showing genomic relations between the *Sitopsis* section *Aegilops* (except *Ae*. *speltoides*) and the wheat.

Supplementary Figure 11 Bar chart showing genomic relation between U genome diploid *Ae*. *umbellulata* and wheat.

Supplementary Figure 12 Bar chart showing genomic relation between N genome diploid *Ae*. *uniaristata* and wheat.

Supplementary Figure 13 Bar chart showing genomic relation between M genome diploid *Ae*. *comosa* and wheat.

Supplementary Figure 14 Bar chart showing genomic relation between C genome diploid *Ae*. *markgraffii* and wheat.

Supplementary Figure 15 Bar charts showing genomic relations between *Ae*. *mutica* and wheat.

Supplementary Table 1 List of *Aegilops* germplasms in the WGRC gene bank collection with the taxa and origins of the accessions (separate excel file).

Supplementary Table 2 Different SNP matrices, population genotyped, the reference sequence used and the application which used the SNP matrix.

Supplementary Table 3 Misclassified and genetically identical (redundant) *Aegilops* accessions (separate excel file).

Supplementary Table 4
*Sitopsis* section *Aegilops* and *Ae*. *mutica* pairwise F_ST_ values.

Supplementary Table 5 Total segregating loci in UM and UX genome species when called variants on the M genome and U genome mock references independently.

## References

[B1] Adhikari L. Lindstrom O. M. Markham J. Missaoui A. M. (2018). Dissecting key adaptation traits in the polyploid perennial medicago sativa using GBS-SNP mapping. Front. Plant Sci. 9. doi: 10.3389/fpls.2018.00934 PMC6039623 30022989

[B2] Adhikari L. Raupp J. Wu S. Wilson D. Evers B. Koo D. H.. (2022a). Genetic characterization and curation of diploid A-genome wheat species. Plant Physiol. 188, 2101–2114. doi: 10.1093/plphys/kiac006 35134208 PMC8968256

[B3] Adhikari L. Shrestha S. Wu S. Crain J. Gao L. Evers B.. (2022b). A high-throughput skim-sequencing approach for genotyping, dosage estimation and identifying translocations. Sci. Rep. 12, 17583. doi: 10.1038/s41598-022-19858-2 36266371 PMC9584886

[B4] Ahmed H. I. Heuberger M. Schoen A. Koo D.-H. Quiroz-Chavez J. Adhikari L.. (2023). Einkorn genomics sheds light on history of the oldest domesticated wheat. Nature 620, 830–838. doi: 10.1038/s41586-023-06389-7 37532937 PMC10447253

[B5] Appels R. Eversole K. Feuillet C. Keller B. Rogers J. Stein N.. (2018). Shifting the limits in wheat research and breeding using a fully annotated reference genome. Science 361. doi: 10.1126/science.aar7191 30115783

[B6] Asseng S. Ewert F. Martre P. Rötter R. P. Lobell D. B. Cammarano D.. (2015). Rising temperatures reduce global wheat production. Nat. Climate Change 5, 143–147. doi: 10.1038/nclimate2470

[B7] Avni R. Lux T. Minz-Dub A. Millet E. Sela H. Distelfeld A.. (2022). Genome sequences of three Aegilops species of the section Sitopsis reveal phylogenetic relationships and provide resources for wheat improvement. Plant J. 110, 179–192. doi: 10.1111/tpj.15664 34997796 PMC10138734

[B8] Badaeva E. Amosova A. Samatadze T. Zoshchuk S. Shostak N. Chikida N.. (2004). Genome differentiation in Aegilops. 4. Evolution of the U-genome cluster. Plant Systematics Evol. 246, 45–76. doi: 10.1007/s00606-003-0072-4

[B9] Badaeva E. D. Friebe B. Zoshchuk S. A. Zelenin A. V. Gill B. S. (1998). Molecular cytogenetic analysis of tetraploid and hexaploid aegilops crassa. Chromosome Res. 6, 629–637. doi: 10.1023/A:1009257527391 10099876

[B10] Badaeva E. D. Ruban A. S. Shishkina A. A. Sibikeev S. N. Druzhin A. E. Surzhikov S. A.. (2018). Genetic classification of Aegilops columnaris Zhuk. (2n=4x=28, UcUcXcXc) chromosomes based on FISH analysis and substitution patterns in common wheat × Ae. columnaris introgressive lines. Genome 61, 131–143. doi: 10.1139/gen-2017-0186%M29216443 29216443

[B11] Bernhardt N. Brassac J. Dong X. Willing E.-M. Poskar C. H. Kilian B.. (2020). Genome-wide sequence information reveals recurrent hybridization among diploid wheat wild relatives. Plant J. 102, 493–506. doi: 10.1111/tpj.14641 31821649

[B12] Chen W.-C. (2011). Overlapping codon model, phylogenetic clustering, and alternative partial expectation conditional maximization algorithm (IA, United States: Iowa State University).

[B13] Chen S. Zhou Y. Chen Y. Gu J. (2018). fastp: an ultra-fast all-in-one FASTQ preprocessor. Bioinformatics 34, i884–i890. doi: 10.1093/bioinformatics/bty560 30423086 PMC6129281

[B14] Cruz C. D. Peterson G. L. Bockus W. W. Kankanala P. Dubcovsky J. Jordan K. W.. (2016). The 2NS Translocation from Aegilops ventricosa Confers Resistance to the Triticum Pathotype of Magnaporthe oryzae. Crop Sci. 56, 990–1000. doi: 10.2135/cropsci2015.07.0410 27814405 PMC5087972

[B15] Dvorak J. (1998). “Genome analysis in the Triticum-Aegilops alliance,” in Proceedings of the 9th international wheat genetics symposium. Slinkard A. E. (Ed.). (Saskatoon, Saskatchewan, Canada: University Extension Press, University of Saskatchewan) 1, 8–11.

[B16] Endelman J. B. (2011). Ridge regression and other kernels for genomic selection with R package rrBLUP. Plant Genome 4, 250–255. doi: 10.3835/plantgenome2011.08.0024

[B17] Friebe B. Jiang J. Raupp W. J. Mcintosh R. A. Gill B. S. (1996). Characterization of wheat-alien translocations conferring resistance to diseases and pests: current status. Euphytica 91, 59–87. doi: 10.1007/BF00035277

[B18] Gao L. Koo D.-H. Juliana P. Rife T. Singh D. Lemes Da Silva C.. (2021). The Aegilops ventricosa 2NvS segment in bread wheat: cytology, genomics and breeding. Theor. Appl. Genet. 134, 529–542. doi: 10.1007/s00122-020-03712-y 33184704 PMC7843486

[B19] Gaurav K. Arora S. Silva P. Sánchez-Martín J. Horsnell R. Gao L.. (2022). Population genomic analysis of Aegilops tauschii identifies targets for bread wheat improvement. Nat. Biotechnol. 40, 422–431. doi: 10.1038/s41587-021-01058-4 34725503 PMC8926922

[B20] Glaubitz J. C. Casstevens T. M. Lu F. Harriman J. Elshire R. J. Sun Q.. (2014). TASSEL-GBS: A high capacity genotyping by sequencing analysis pipeline. PloS One 9, e90346. doi: 10.1371/journal.pone.0090346 24587335 PMC3938676

[B21] Glémin S. Scornavacca C. Dainat J. Burgarella C. Viader V. Ardisson M.. (2019). Pervasive hybridizations in the history of wheat relatives. Sci. Adv. 5, eaav9188. doi: 10.1126/sciadv.aav9188 31049399 PMC6494498

[B22] Grewal S. Coombes B. Joynson R. Hall A. Fellers J. Yang C. Y.. (2022). Chromosome-specific KASP markers for detecting Amblyopyrum muticum segments in wheat introgression lines. Plant Genome 15, e20193. doi: 10.1002/tpg2.20193 35102721 PMC12807185

[B23] Haudry A. Cenci A. Ravel C. Bataillon T. Brunel D. Poncet C.. (2007). Grinding up wheat: A massive loss of nucleotide diversity since domestication. Mol. Biol. Evol. 24, 1506–1517. doi: 10.1093/molbev/msm077 17443011

[B24] Jones J. K. Majisu B. N. (1968). THE HOMOEOLOGY OF AEGILOPS MUTICA CHROMOSOMES. Can. J. Genet. Cytology 10, 620–626. doi: 10.1139/g68-080

[B25] Kilian B. Mammen K. Millet E. Sharma R. Graner A. Salamini F.. (2011). “Aegilops,” in Wild Crop Relatives: Genomic and Breeding Resources: Cereals. Ed. Kole C. (Berlin, Heidelberg: Springer Berlin Heidelberg).

[B26] Kishii M. (2019). An update of recent use of aegilops species in wheat breeding. Front. Plant Sci. 10. doi: 10.3389/fpls.2019.00585 PMC6521781 31143197

[B27] Koo D.-H. Liu W. Friebe B. Gill B. S. (2017). Homoeologous recombination in the presence of Ph1 gene in wheat. Chromosoma 126, 531–540. doi: 10.1007/s00412-016-0622-5 27909815

[B28] Leigh F. J. Wright T. I. C. Horsnell R. A. Dyer S. Bentley A. R. (2022). Progenitor species hold untapped diversity for potential climate-responsive traits for use in wheat breeding and crop improvement. Heredity 128, 291–303. doi: 10.1038/s41437-022-00527-z 35383318 PMC9076643

[B29] Li H. (2011). A statistical framework for SNP calling, mutation discovery, association mapping and population genetical parameter estimation from sequencing data. Bioinformatics 27, 2987–2993. doi: 10.1093/bioinformatics/btr509 21903627 PMC3198575

[B30] Li H. Dong Z. Ma C. Tian X. Xiang Z. Xia Q.. (2019). Discovery of powdery mildew resistance gene candidates from Aegilops biuncialis chromosome 2Mb based on transcriptome sequencing. PloS One 14, e0220089. doi: 10.1371/journal.pone.0220089 31710598 PMC6844473

[B31] Li L. F. Zhang Z. B. Wang Z. H. Li N. Sha Y. Wang X. F.. (2022). Genome sequences of five Sitopsis species of Aegilops and the origin of polyploid wheat B subgenome. Mol. Plant 15, 488–503. doi: 10.1016/j.molp.2021.12.019 34979290

[B32] Lopes M. S. El-Basyoni I. Baenziger P. S. Singh S. Royo C. Ozbek K.. (2015). Exploiting genetic diversity from landraces in wheat breeding for adaptation to climate change. J. Exp. Bot. 66, 3477–3486. doi: 10.1093/jxb/erv122 25821073

[B33] Luo M.-C. Deal K. R. Yang Z.-L. Dvorak J. (2005). Comparative genetic maps reveal extreme crossover localization in the Aegilops speltoides chromosomes. Theor. Appl. Genet. 111, 1098–1106. doi: 10.1007/s00122-005-0035-y 16088396

[B34] Luo M.-C. Gu Y. Q. Puiu D. Wang H. Twardziok S. O. Deal K. R.. (2017). Genome sequence of the progenitor of the wheat D genome Aegilops tauschii. Nature 551, 498–502. doi: 10.1038/nature24486 29143815 PMC7416625

[B35] Marais G. F. Mccallum B. Snyman J. E. Pretorius Z. A. Marais A. S. (2005). Leaf rust and stripe rust resistance genes Lr54 and Yr37 transferred to wheat from Aegilops kotschyi. Plant Breed. 124, 538–541. doi: 10.1111/j.1439-0523.2005.01116.x

[B36] Melo A. T. O. Bartaula R. Hale I. (2016). GBS-SNP-CROP: a reference-optional pipeline for SNP discovery and plant germplasm characterization using variable length, paired-end genotyping-by-sequencing data. BMC Bioinf. 17, 29. doi: 10.1186/s12859-016-0879-y PMC4709900 26754002

[B37] Nei M. (1987). Molecular Evolutionary Genetics (New York: Columbia University Press), 512.

[B38] Paradis E. Schliep K. (2019). ape 5.0: an environment for modern phylogenetics and evolutionary analyses in R. Bioinformatics 35, 526–528. doi: 10.1093/bioinformatics/bty633 30016406

[B39] Poland J. A. Brown P. J. Sorrells M. E. Jannink J.-L. (2012). Development of high-density genetic maps for barley and wheat using a novel two-enzyme genotyping-by-sequencing approach. PloS One 7, e32253. doi: 10.1371/journal.pone.0032253 22389690 PMC3289635

[B40] R Core Team. (2020). R: A Language and Environment for Statistical Computing. Vienna, Austria: R Foundation for Statistical Computing. Available at: https://www.r-project.org/.

[B41] Raj A. Stephens M. Pritchard J. K. (2014). fastSTRUCTURE: variational inference of population structure in large SNP data sets. Genetics 197, 573–589. doi: 10.1534/genetics.114.164350 24700103 PMC4063916

[B42] Rakszegi M. Molnár I. Darkó É. Tiwari V. K. Shewry P. (2020). Editorial: aegilops: promising genesources to improve agronomical and quality traits of wheat. Front. Plant Sci. 11. doi: 10.3389/fpls.2020.01060 PMC7371959 32760415

[B43] Resta P. Zhang G.-B. Dubcovsky J. Dvořák J. (1996). The origins of the genomes of Triticum biunciale, t. ovatum, t. neglectum, t. columnare, and t. rectum (poaceae) based on variation in repeated nucleotide sequences. Am. J. Bot. 83, 1556–1565. doi: 10.1002/j.1537-2197.1996.tb12813.x

[B44] Said M. Holušová K. Farkas A. Ivanizs L. Gaál E. Cápal P.. (2021). Development of DNA Markers From Physically Mapped Loci in Aegilops comosa and Aegilops umbellulata Using Single-Gene FISH and Chromosome Sequences. Front. Plant Sci. 12. doi: 10.3389/fpls.2021.689031 PMC8240756 34211490

[B45] Saripalli G. Adhikari L. Amos C. Kibriya A. Ahmed H. I. Heuberger M.. (2023). Integration of genetic and genomics resources in einkorn wheat enables precision mapping of important traits. Commun. Biol. 6, 835. doi: 10.1038/s42003-023-05189-z 37573415 PMC10423216

[B46] Singh N. Wu S. Raupp W. J. Sehgal S. Arora S. Tiwari V.. (2019a). Efficient curation of genebanks using next generation sequencing reveals substantial duplication of germplasm accessions. Sci. Rep. 9, 650. doi: 10.1038/s41598-018-37269-0 30679756 PMC6346010

[B47] Singh N. Wu S. Tiwari V. Sehgal S. Raupp J. Wilson D.. (2019b). Genomic analysis confirms population structure and identifies inter-lineage hybrids in aegilops tauschii. Front. Plant Sci. 10. doi: 10.3389/fpls.2019.00009 PMC6357674 30740115

[B48] Suneja Y. Gupta A. K. Bains N. S. (2019). Stress Adaptive Plasticity: Aegilops tauschii and Triticum dicoccoides as Potential Donors of Drought Associated Morpho-Physiological Traits in Wheat. Front. Plant Sci. 10. doi: 10.3389/fpls.2019.00211 PMC6397871 30858862

[B49] Tanaka S. Yoshida K. Sato K. Takumi S. (2020). Diploid genome differentiation conferred by RNA sequencing-based survey of genome-wide polymorphisms throughout homoeologous loci in Triticum and Aegilops. BMC Genomics 21, 246. doi: 10.1186/s12864-020-6664-3 32192452 PMC7083043

[B50] Van Slageren M. W. (1994). Wild wheats: a monograph of Aegilops L. and Amblyopyrum (Jaub. & Spach) Eig (Poaceae) (the Netherlands: Wageningen Agricultural University Papers).

[B51] Volk G. M. Byrne P. F. Coyne C. J. Flint-Garcia S. Reeves P. A. Richards C. (2021). Integrating genomic and phenomic approaches to support plant genetic resources conservation and use. Plants 10, 2260. doi: 10.3390/plants10112260 34834625 PMC8619436

[B52] Waines J. G. Barnhart D. (1992). Biosystematic research in aegilops and triticum. Hereditas 116, 207–212. doi: 10.1111/j.1601-5223.1992.tb00825.x

[B53] Wang L. Zhu T. Rodriguez J. C. Deal K. R. Dubcovsky J. Mcguire P. E.. (2021). Aegilops tauschii genome assembly Aet v5.0 features greater sequence contiguity and improved annotation. G3 (Bethesda) 11. doi: 10.1093/g3journal/jkab325 PMC8664484 34515796

[B54] Yu G. Matny O. Champouret N. Steuernagel B. Moscou M. J. Hernández-Pinzón I.. (2022). Aegilops sharonensis genome-assisted identification of stem rust resistance gene Sr62. Nat. Commun. 13, 1607. doi: 10.1038/s41467-022-29132-8 35338132 PMC8956640

[B55] Zhao C. Liu B. Piao S. Wang X. Lobell D. B. Huang Y.. (2017). Temperature increase reduces global yields of major crops in four independent estimates. Proc. Natl. Acad. Sci. 114, 9326–9331. doi: 10.1073/pnas.1701762114 28811375 PMC5584412

[B56] Zhu T. Wang L. Rimbert H. Rodriguez J. C. Deal K. R. De Oliveira R.. (2021). Optical maps refine the bread wheat Triticum aestivum cv. Chinese Spring genome assembly. Plant J. Cell Mol. Biol. 107, 303–314. doi: 10.1111/tpj.15289 PMC8360199 33893684

[B57] Zohary D. Imber D. (1963). Genetic dimorphism in fruit types in Ægilops speltoides. Heredity 18, 223–231. doi: 10.1038/hdy.1963.24

