# Supplementary material for: Genomic characterization and gene bank curation of Aegilops: the wild relatives of wheat
Source: Front Plant Sci. 2023 Oct 17;14:1268370. doi: 10.3389/fpls.2023.1268370 (PMC10616851; doi:10.3389/fpls.2023.1268370)
Supplement: Supplementary Figure 1 — The GBS SNP–based unrooted neighbor-joining (NJ) tree separating tetraploid and hexaploid accessions of Ae. neglecta (blue clade) and the chromosome counts of two representative individuals from each 4X and 6X sub-clade of the Ae. neglecta. [file DataSheet_1.docx]

**Supplementary Data**

**Genomic Characterization and Gene Bank Curation of *Aegilops*: The Wild Relatives of Wheat**

Laxman Adhikari^1,2^, John Raupp^2^, Shuangye Wu^2^, Dal-Hoe Koo^2^, Bernd Friebe^2^, and Jesse Poland^1,2,3^*

^1^ KAUST Center for Desert Agriculture, Biological and Environmental Science and Engineering Division, King Abdullah University of Science and Technology (KAUST), Thuwal, 23955-6900, Kingdom of Saudi Arabia

^2^ Wheat Genetics Resource Center, Department of Plant Pathology, Kansas State University, Manhattan Kansas, USA, 66502

^3^ Plant Science Program, Biological and Environmental Science and Engineering Division, King Abdullah University of Science and Technology (KAUST), Thuwal, 23955-6900, Kingdom of Saudi Arabia

* Corresponding author: [jesse.poland@kaust.edu.sa](mailto:jesse.poland@kaust.edu.sa) ; ORCID: 0000-0002-7856-1399

**Supplementary Figures**

**Supplementary Figure S1.** The GBS SNP based unrooted neighbor-joining (NJ) tree separating tetraploid and hexaploid accessions of *Ae*. *neglecta* (blue clade) and the chromosome counts of two representative individuals from each 4X and 6X sub-clade of the *Ae*. *neglecta*. The other sister species *Ae*. *columnaris* accessions are shown in the red-colored clade.

**Supplementary Figure S2.** An unrooted neighbor-joining (NJ) tree of *Ae*. *juvenalis*, *Ae*. *crassa and Ae*. *vavilovii.* The tree branches were colored based on the accession’s taxon. The *Ae. crassa* branches we annotated as 4x or 6x based on cytological chromosome counting. The grouping clearly separated 6X and 4X *Ae*. *crassa*.

**Supplementary Figure S3.** Principal component analysis (PCA) plot showing two forms of *Ae*. *speltoides*; var. *speltoides* and *ligustica*. No separate PCA cluster was observed for each of the groups.

**Supplementary Figure S4.** An unrooted neighbor-joining (NJ) tree separating some tetraploid *Aegilops* accessions containing two species whose genome formula is controversial, the *Ae*. *neglecta* and *Ae*. *columnaris*. These two species clustered in the middle of UM genome clade and UC genome clade. The M genome (now proposed X) was a part of traditional *Ae*. *neglecta* and *Ae*. *columnaris* genome formula.

**Supplementary Figure S5.** The bar chart showing the overall sequence read alignment of four tetraploid *Aegilops* species: *Ae. biuncialis, Ae*. *geniculata, Ae*. *columnaris* and *Ae*. *neglecta* when aligned on M and U genome *de* *novo* mock reference.

**Supplementary Figure S6.** Minor allele frequency (MAF) distribution within the loci for the entire *Aegilops* collection. Most of the loci had a very low MAF.

**Supplementary Figure S7.** Distribution of minor alleles frequency (MAF) for segregating variants in *Ae*. *speltoides*.

**Supplementary Figure S8.** An unrooted neighbor-joining (NJ) tree constructed using the genotyping information generated by using wheat B genome as a reference (left); and the unrooted NJ tree constructed using genotyping profile generated using the wheat D genome as a reference (right). The clades were colored based on genetic clustering.

**Supplementary Figure S9.** Bar charts showing genomic relations between the *Sitopsis* section *Aegilops* (except *Ae*. *speltoides*) and the wheat. The number of reads coverage (mapped per 1 Mb wheat genome) are shown for all wheat chromosomes. *Ae*. *sharonensis* and *Ae*. *longissima* appeared as highly genetically similar so their sequences were mapped together.

**Supplementary Figure S10.** Bar charts showing genomic relations between the *Sitopsis* section *Aegilops* (except *Ae*. *speltoides*) and the wheat. The number of variants (# SNPs) identified per wheat chromosome for the four species are shown. *Ae*. *sharonensis* and *Ae*. *longissima* appeared as highly genetically similar so their SNPs were called together.

**Supplementary Figure S11.** Bar chart showing genomic relation between U genome diploid *Ae*. *umbellulata* and wheat. The average number of *Ae*. *umbellulata* sequence reads mapped per Mb of the wheat genome (upper panel), and numbers of *Ae*. *umbellulata* variants mapped on the respective wheat chromosomes (lower panel).

**Supplementary Figure S12.** Bar chart showing genomic relation between N genome diploid *Ae*. *uniaristata* and wheat. The average number of *Ae*. *uniaristata* sequence reads mapped per Mb of the wheat genome (upper panel), and numbers of *Ae*. *uniaristata* variants mapped on the respective wheat chromosomes (lower panel).

**Supplementary Figure S13.** Bar chart showing genomic relation between M genome diploid *Ae*. *comosa* and wheat. The average number of *Ae*. *comosa* sequence reads mapped per Mb of the wheat genome (upper panel), and numbers of *Ae*. *comosa* variants mapped on the respective wheat chromosomes (lower panel).

**Supplementary Figure S14.** Bar chart showing genomic relation between C genome diploid *Ae*. *markgraffii* and wheat. The average number of *Ae*. *markgraffii* sequence reads mapped per Mb of the wheat genome (upper panel), and numbers of *Ae*. *markgraffii* variants mapped on the respective wheat chromosomes (lower panel).

**Supplementary Figure S15.** Bar charts showing genomic relations between *Ae*. *mutica* and wheat. The average number of *Ae*. *mutica* sequence reads mapped per Mb of the wheat genome (upper panel), and numbers of *Ae*. *mutica* variants mapped on the respective wheat chromosomes (lower panel).

**Supplementary Tables**

**Supplementary Table S1**. List of *Aegilops* germplasms in the WGRC gene bank collection with the taxa and origins of the accessions.

[external file]

**Supplementary Table S2**. Different SNP matrices, population genotyped, the reference sequence used and the application which used the SNP matrix.

| **Species** | **Total Accessions** | **Reference/Mock Reference genome Source Species** | **Total Filtered SNPs** | **Analysis** |
| --- | --- | --- | --- | --- |
| All 23 species | 1051 | *De* *novo*: derived from sequence reads representing all *Aegilops* genomes | 54667 | Gbc, PA, GR |
| All 23 species | 1041 | After filtration: (MAF > 0.01, missing <30%, heterozygosity <10%) | 46879 | Gbc, PA, GR |
| All U genome species | 596 | *De* *novo*: from *Ae*. *umbellulata* sequence reads | 18653 | Gbc, PA, GR |
| *Ae*. *neglecta* and *Ae*. *columnaris* | 88 | *De* *novo*: from *Ae*. *neglecta and Ae. columnaris* sequence reads | 21035 | Gbc |
| *Ae*. *umbellulata* (U) | 58 | *De* *novo*: from *Ae*. *umbellulata* sequence reads | 18653 | GR |
| *Ae*. *comosa* (M) | 17 | *De* *novo*: from Ae. comosa sequence reads | 23304 | GR |
| S-genome diploids and *Ae*. *mutica* | 214 | *Ae*. *speltoides* reference genome | 13553 | Gbc, PA |
| *Ae*. *searsii* | 18 | *Ae*. *searsii* reference genome | 11663 | Gbc |
| *Ae*. *sharonesis* and  *Ae*. *longissima* | 23 | *Ae*. *sharonensis* reference genome | 19000 | Gbc |
| *Ae*. *bicornis* | 13 | *Ae*. *bicornis* reference genome |  | Gbc |
| *Ae*. *tauschii* | 47 | *Ae*. *tauschii* reference genome | 28617 | Gbc |
| *Ae*. *speltoides* | 97 | Chinese spring wheat reference genome | 23281 | GR |
| *Ae*. *searsii* | 18 | Chinese spring wheat reference genome | 7114 | GR |
| *Ae*. *sharonesis* and  *Ae*. *longissima* | 23 | Chinese spring wheat reference genome | 8192 | GR |
| *Ae*. *bicornis* | 13 | Chinese spring wheat reference genome | 2570 | GR |
| *Ae*. *mutica* | 54 | Chinese spring wheat reference genome | 17638 | GR |
| Ae. *umbellulata* | 58 | Chinese spring wheat reference genome | 4048 | GR |
| *Ae*. *uniaristata* | 24 | Chinese spring wheat reference genome | 1142 | GR |
| *Ae*. *comosa* | 17 | Chinese spring wheat reference genome | 2853 | GR |
| *Ae*. *markgraffii* | 16 | Chinese spring wheat reference genome | 3874 | GR |

Gbc = Gene bank curation

PA = Population analysis

GR = Genome relation

**Supplementary Table S3**. Misclassified and genetically identical (redundant) *Aegilops* accessions. The redundant accession was determined for only those diploid species collections whose genotyping was based on the variants called on their reference genome.

[external file]

**Supplementary Table S4.** *Sitopsis* section *Aegilops* and *Ae*. *mutica* pairwise F_ST_ values. The higher values indicate more genetic differentiation and lower values indicate a lower genetic differentiation between pairs.

|  | *Ae*. *longissima* | *Ae*. *searsii* | *Ae*. *sharonensis* | *Ae*. *mutica* | *Ae*. *speltoides* |
| --- | --- | --- | --- | --- | --- |
| *Ae*. *bicornis* | 0.027 | 0.057 | 0.027 | 0.073 | 0.085 |
| *Ae*. *longissima* |  | 0.054 | 0.006 | 0.067 | 0.079 |
| *Ae*. *searsii* |  |  | 0.055 | 0.075 | 0.088 |
| *Ae*. *sharonensis* |  |  |  | 0.068 | 0.081 |
| *Ae*. *mutica* |  |  |  |  | 0.065 |

**Supplementary Table S5**. Total segregating loci in UM and UX genome species when called variants on the M genome and U genome mock references independently.

| **Species** | **No. of individuals** | **Segregating loci**  **M-genome variants** | **Segregating loci**  **U-genome variants** |
| --- | --- | --- | --- |
| *Ae*. *comosa* | 17 | 23304 | - |
| *Ae*. *biuncials* (UM) | 53 | 11598 (50%) | 15380 (83%) |
| *Ae*. *geniculata* (MU) | 143 | 10590 (46%) | 11331 (61%) |
| *Ae*. *columnaris* (UX) | 12 | 2287 (10%) | 9849 (53%) |
|  |  |  |  |
| *Ae*. *neglecta* (UX, UXN) | 76 | 5467 (24%) | 12013 (65%) |
| *Ae*. *umbellulata* (U) | 58 | - | 18653 |
